# Serum metabolomic signatures of vegetarians relative to omnivores in a Chinese cohort: associations with cardiometabolic risk factors

**DOI:** 10.3389/fnut.2025.1672143

**Published:** 2025-09-23

**Authors:** Die Yao, Linxi Qian, Yizhou Jin, Xiaodi Wang, Xintong Lu, Fangfang Song, Xiuhua Shen

**Affiliations:** ^1^Department of Clinical Nutrition, Xinhua Hospital Affiliated to Shanghai Jiao Tong University School of Medicine, Shanghai, China; ^2^Department of Clinical Nutrition, College of Health Science and Technology, Shanghai Jiao Tong University School of Medicine, Shanghai, China; ^3^Shanghai Key Laboratory of Pediatric Gastroenterology and Nutrition, Xinhua Hospital Affiliated to Shanghai Jiao Tong University School of Medicine, Shanghai, China; ^4^School of Public Health, Shanghai Jiao Tong University School of Medicine, Shanghai, China

**Keywords:** vegetarian diets, omnivorous diets, metabolomics, cardiometabolic risk factors, dietary intake

## Abstract

**Introduction:**

This study aimed to compare serum metabolomic profiles between vegetarians and omnivores in a Chinese cohort and investigate their associations with cardiometabolic risk factors, including obesity, blood pressure, lipid profiles, and glucose metabolism.

**Materials and methods:**

A cross-sectional study included 444 participants (222 vegetarians and 222 omnivores) matched by age and sex. Serum metabolomic profiling was performed using ultra-performance liquid chromatography–tandem mass spectrometry. Correlation analyses and multivariate linear regression models were employed to examine the associations between metabolites and cardiometabolic risk factors, adjusting for potential confounders such as age, sex, physical activity, and dietary patterns.

**Results:**

Seventeen key differential metabolites were identified, with 11 upregulated (e.g., maleic acid, methylcysteine, citric acid, indolepropionic acid [IPA]) and 6 downregulated (e.g., docosahexaenoic acid, eicosapentaenoic acid, creatine) in vegetarians compared to omnivores. After adjusting for covariates, metabolites such as methylcysteine, aconitic acid, and IPA were inversely associated with obesity indices (BMI, waist-to-hip ratio, body fat percentage), blood pressure, and lipid profiles, while creatine showed positive associations with obesity markers. Notably, IPA was linked to reduced systolic and diastolic blood pressure, and aconitic acid correlated with improved insulin sensitivity. Dietary analysis revealed that IPA and methylcysteine were positively associated with plant-based foods such as whole grains, millet, and legumes, while docosahexaenoic acid and eicosapentaenoic acid showed strong positive correlations with animal-based foods, particularly seafood.

**Conclusion:**

Vegetarian diets are associated with unique serum metabolomic profiles that may improve cardiometabolic health.

## Graphical abstract

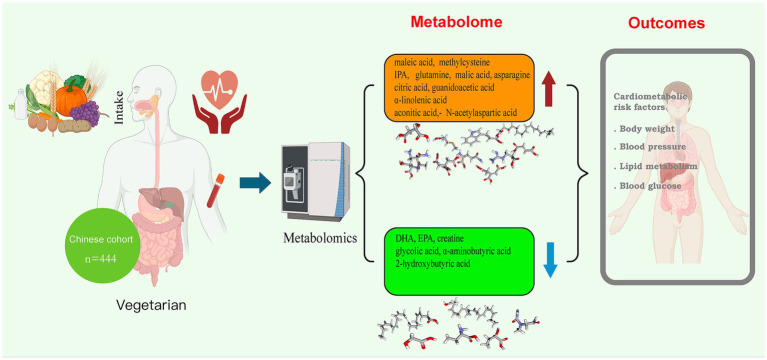

## 1 Introduction

Vegetarian diets, defined by the exclusion of meat and varying degrees of other animal products, have evolved from historical roots in ethics and religion to a modern dietary strategy embraced for its health and environmental benefits (1). Accumulating evidence highlights the association of vegetarian diets with reduced risks of cardiometabolic diseases, including obesity, type 2 diabetes, dyslipidemia, and cardiovascular disorders (2–4). While mechanistic explanations often focus on dietary fiber, antioxidants, and reduced saturated fat intake (5–7), emerging research underscores the importance of systemic metabolic adaptations in mediating these benefits (8). However, the specific metabolic pathways modulated by vegetarian diets—particularly in non-Western populations—remain underexplored, limiting the translation of findings into culturally tailored dietary recommendations.

Metabolomics, the comprehensive analysis of small-molecule metabolites in biological systems, has emerged as a powerful tool to decode the dynamic interplay between diet and physiology (9). Unlike other omics approaches, metabolomics captures real-time metabolic responses to dietary exposures, offering insights into mechanisms linking diet to health outcomes (10). Prior studies comparing vegetarians and non-vegetarians have identified distinct metabolic profiles, including altered levels of amino acids (e.g., essential amino acids), lipid species (e.g., several fatty acids), and microbiota-derived metabolites (e.g., short-chain fatty acids) (11–13). These findings suggest that vegetarian diets may modulate pathways related to energy metabolism, inflammation, and gut microbiome activity. However, few studies have systematically integrated vegetarian metabolomic data with detailed cardiometabolic phenotyping, hindering the identification of clinically actionable biomarkers.

In China, rapid urbanization and dietary transitions have precipitated a dual burden of undernutrition and rising cardiometabolic diseases (14). Traditional Chinese diets rich in plant-based foods, are increasingly supplanted by meat-centric eating patterns, mirroring global trends. This shift underscores the urgency to understand how dietary transitions impact metabolic health in this population. Existing metabolomic studies in Chinese populations have predominantly focused on disease-specific biomarkers (e.g., diabetes, hypertension) (15–17) or isolated nutrient effects (e.g., vitamin D, *ω*-3 fatty acids) (18, 19), with no published research specifically examining serum metabolomic profiles associated with vegetarian diets. This gap is particularly critical given the unique dietary components (e.g., soy products, rice, and wheat-based foods) and cooking practices in Chinese cuisine, which may drive distinct metabolic adaptations compared to Western vegetarian diets. The absence of such data limits the translation of global vegetarian diet research into actionable insights for Chinese populations.

To address these limitations, we conducted a cross-sectional study to compare serum metabolomic profiles of Chinese vegetarians and omnivores using targeted metabolomics. Our study aims to (1) identify differential metabolites associated with vegetarian diets, (2) evaluate their associations with cardiometabolic risk factors (e.g., obesity indices, blood pressure, lipid profiles, glucose homeostasis), and (3) explore correlations between metabolites and dietary intake patterns. Rigorous adjustments for potential confounders, such as age, sex, and physical activity, were implemented to isolate diet-specific metabolic signatures.

## 2 Materials and methods

### 2.1 Study population

**Figure 1:**
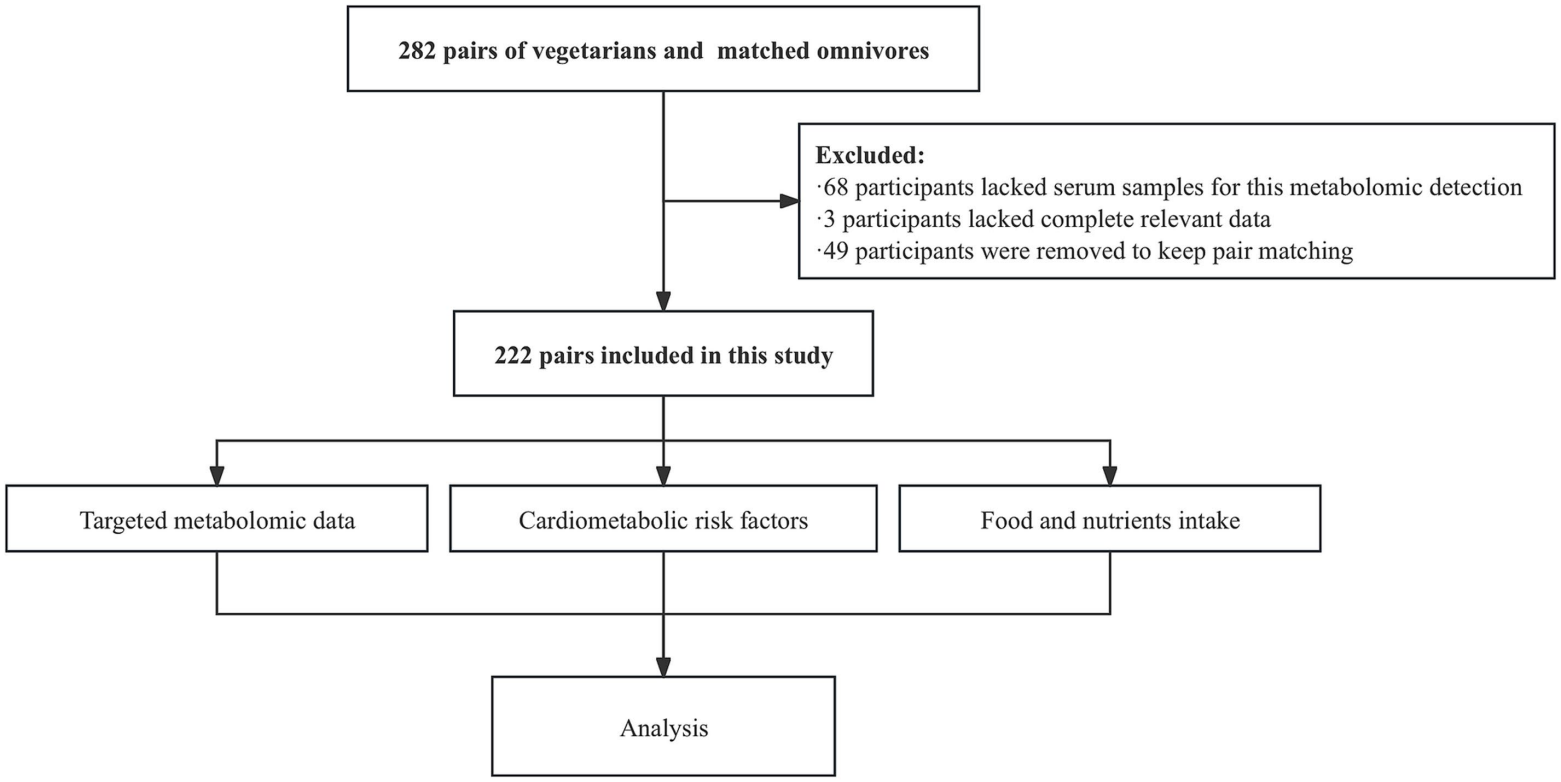
Participants flowchart.

Healthy vegetarians were recruited through vegetarian associations and restaurants in Shanghai from March to May 2016. Inclusion criteria required participants to: (1) be aged 18 years or older; (2) have resided in Shanghai for at least 6 months; (3) have maintained a vegetarian diet for a minimum of 1 year; and (4) be able to comprehend the questionnaire content. Exclusion criteria included: (1) a history of severe nutritional malabsorption or systemic diseases; and (2) pregnancy or breastfeeding within the previous 12 months. Omnivore participants were recruited from the friends and relatives of the vegetarians and were matched by sex and age (±1 year). In total, 282 pairs of vegetarians and omnivores were recruited, all of whom reported no history of diabetes or metabolic diseases. For this serum metabolomics study, 222 pairs (444 subjects) with serum samples and complete relevant data of cardiometabolic risk factors were included (Figure 1). This study was approved by the Institutional Review Board of the Shanghai Jiao Tong University School of Medicine (No. 2016029). Informed consent was obtained from all subjects involved in the study.

### 2.2 Demographic data and dietary assessment

Questionnaires were administered to participants, collecting demographic and individual behavioral information, such as age, sex, income, alcohol consumption, smoking, physical activity, sedentary time, sleep quality, vegetarian pattern, and vegetarian duration. Sleep quality was evaluated by the Chinese version of the Pittsburg Sleep Questionnaire Index (PSQI). Experienced dietitians administered a face-to-face semi-quantitative food frequency questionnaire (FFQ) to all participants to assess the intake amount and frequency of various foods over the preceding year. The questionnaire from the 2002 China Nutrition and Health Survey was adopted, encompassing 112 food categories, which were organized into 13 food modules: grains and tubers, beans, vegetables, fungi and algae, fruits, dairy, eggs, nuts, beverages, meat, oils, snacks, and condiments. Participants who adhered to a vegetarian diet at all meals daily for at least 1 year were classified as vegetarian. Otherwise, they were categorized as omnivores. To enhance the accuracy of participants’ food intake estimates, food pictures and models were employed. Daily nutrient intakes were calculated from the questionnaire using Nutrition Calculator v2.5 software, which was developed by the National Institute for Nutrition and Health of the Chinese Centre for Disease Control and Prevention, in collaboration with Beijing Feihua Communication Technology Co., LTD.

### 2.3 Anthropometric and biochemical measurements

The height, weight, waist circumference, hip circumference, body composition, and blood pressure of the participants were measured by experienced dietitians. Body mass index (BMI) was calculated using the formula: weight (kg) /height (m^2^), and the waist-to-hip ratio (WHR) was determined by dividing waist circumference (cm) by hip circumference (cm). Body composition was assessed with a calibrated bioimpedance device (InBody720, Biospace Inc., Korea), which provided the percent body fat (PBF). Systolic blood pressure (SBP) and diastolic blood pressure (DBP) were measured using an UA-774 Aiander electronic sphygmomanometer.

Blood samples were collected following at least 8 h of overnight fasting using Gel & Clot Activator tubes for venous blood collection from Wenzhou GAODE Medical Instrument Co., LTD. The biochemical markers measured included total cholesterol (TC), triglycerides (TG), low-density lipoprotein cholesterol (LDL-C), high-density lipoprotein cholesterol (HDL-C), fasting blood glucose (FG), and fasting insulin (FI). Homeostasis model assessment of insulin resistance (HOMA-IR) and *β*-cell function (HOMA-β) were calculated using FG and FI (20). Biochemical analyses were performed by the Clinical Laboratory Center at Shanghai Xinhua Hospital.

### 2.4 Sample preparation and instrumental analysis

For targeted metabolomic profiling of serum samples, the Q300 platform (Human Metabolomics Institute, Inc., China) was utilized, as described in previous studies with minor modification (21). The method was optimized for high-throughput detection and quantification of 306 metabolites. In brief, a 20 μL aliquot of serum was mixed with 120 μL methanol containing most of the internal standards in a 96-well plate. The mixture was vortexed for 10 min and then centrifuged at 4°C at 4000 g for 30 min. A 30 μL aliquot of the supernatant was transferred to another 96-well plate for further derivatization. After incubating at 30°C for 60 min, the reaction was terminated by adding 400 μL of a 50% methanol solution and centrifuged. The resulting 140 μL supernatant was transferred to a new 96-well plate, and 10 μL of derivatized internal standard for short-chain fatty acids was added, followed by mixing and centrifugation.

Analyses were performed using a Waters ACQUITY ultraperformance liquid chromatograph coupled with an XEVO TQ-S mass spectrometer, both controlled by MassLynx 4.1 software (Waters, United States). Chromatographic separation was conducted on an ACQUITY BEH C18 column (1.7 μm, 100 mm × 2.1 mm) (Waters). The mobile phase consisted of water with 0.1% formic acid (A) and acetonitrile/isopropanol (70:30, v/v) (B). The gradient elution program was as follows: 0–1 min (5% B), 1–5 min (5–30% B), 5–9 min (30–50% B), 9–12 min (50–78% B), 12–15 min (78–95% B), 15–16 min (95–100% B), 16–18 min (100% B), 18–18.1 min (100–5% B), 18.1–20 min (5% B), with a flow rate of 0.4 mL/min. The mass spectrometer was operated in both positive and negative ion modes, with a capillary voltage of 1.2 kV for negative mode and 3.2 kV for positive mode, a source temperature of 150°C, a desolvation temperature of 550°C, and a desolvation gas flow rate of 1,200 L/h.

### 2.5 Metabolomic data analysis

The UPLCTQMS data were processed using a TMBQ software (v1.0) to perform peak integration, calibration, and quantification of the metabolites. Briefly, the compounds were identified via the molecular weight and retention time of reference standards, calibrated by internal standards, and quantified by the standard curve generated via a series of diluted reference standards solution. Missing data were handled by replacing undetected metabolites, assumed to be below the LOD, with one Nth of the minimum detected value, where *N* is the sample size. Principal component analysis (PCA), partial least square discriminant analysis (PLS-DA) and orthogonal partial least-squares-discriminant analysis (OPLS-DA) were conducted based on the metabolite profile. The variable importance in the projection (VIP) values obtained from the OPLS-DA model were taken as a criterion for differential metabolites selection. The OPLS-DA model was further verified by a permutation test to avoid transition fit of the model. For univariate testing, either paired *t*-tests or Wilcoxon signed-rank tests were employed to compare between groups, depending on data normality and homoscedasticity. Differential metabolites were selected in the first round based on VIP > 1 and *p* < 0.01. In the second round, the selection criteria were VIP > 1.8 and *p* < 1e-08. The combined results of OPLS-DA and univariate analysis from the first and second rounds of screening were classified as crude and fine differential metabolites, respectively. The differential metabolites were then imported into the human hsa database of the Kyoto Encyclopedia of Genes and Genomes (KEGG) for further pathway analysis.

### 2.6 Statistical analysis

Descriptive statistics were calculated to summarize population characteristics. Continuous variables were expressed as means ± standard deviations (SD), while categorical variables were presented as frequencies and percentages. Comparative analyses of population characteristics between vegetarians and omnivores were performed using appropriate statistical tests: continuous variables were analyzed using paired *t*-tests or Wilcoxon rank-sum tests based on normality assumptions, and categorical variables were examined using McNemar tests. The selection of differential metabolites and the comparison of these metabolites between dietary groups are described separately in the metabolomic data analysis section.

Spearman correlation analyses were employed to examine the relationships between differential metabolite concentrations and dietary components, including foods and nutrients, across the entire cohort as well as within the vegetarian and omnivore subgroups. To investigate the associations between differential metabolites and cardiometabolic risk factors, metabolite concentrations were log-transformed (Ln) to achieve normal distribution. Partial correlation analyses were first performed to assess these associations. Following this, multivariate linear regression was used to further examine these relationships. In multivariate linear regression analyses, participants were categorized into three groups based on tertiles of metabolite concentrations (low, medium, and high), with the low concentration group (T1) serving as the reference category. *β* coefficients and 95% confidence intervals (CIs) were calculated for the medium (T2) and high (T3) concentration groups. Additionally, the median concentration of each differential metabolite within each tertile was treated as a continuous variable to perform trend tests. To control for potential confounders in the analysis of cardiometabolic risk factors, adjustments were made for both partial correlation analysis and multivariate linear regression as follows: for obesity indicators, the models accounted for age, sex, exercise time, alcohol consumption, and dietary pattern; for other cardiometabolic risk factors, BMI was additionally included as a covariate. All statistical analyses were conducted using SPSS version 26.0 software (SPSS Inc., United States) and R software version 4.2.1 (R Foundation for Statistical Computing, Austria), and statistical significance was defined as a two-sided *p* value of less than 0.05.

## 3 Results

### 3.1 Characteristics of the study population

This study included 444 participants, consisting of 222 vegetarians and 222 omnivores, who were matched by gender and age. The mean ages of vegetarians and omnivores were 34.88 ± 8.47 years and 34.35 ± 8.59 years, respectively. Among the participants, 183 pairs (82.4%) were female. Of the 222 vegetarians, 58 (26.1%) were vegans, while 164 (73.9%) were lacto-ovo vegetarians. The average duration of adherence to a vegetarian diet exceeded 5 years. Demographic characteristics and levels of cardiometabolic risk indicators are summarized in Table 1. Regarding demographic variables, no significant differences were found between vegetarians and omnivores in terms of education, income, smoking, exercise habits, and PSQI sleep scores. However, the proportion of nondrinkers was significantly higher in the vegetarian group compared to the omnivore group (*p* < 0.001). In terms of cardiometabolic risk factors, vegetarians exhibited lower BMI, WHR, PBF, SBP, TC, HDL-C, LDL-C, FG, FI, and HOMA-IR scores compared to omnivores (*p* < 0.001). However, there were no significant differences in DBP, TG, and HOMA-β between the two groups.

**Table 1:** Characteristics of vegetarians and omnivores.

	Vegetarians (*n* = 222)	Omnivores (*n* = 222)	*p*
Female, *n* (%)	183 (82.4)	183 (82.4)	
Age (years)	34.88 ± 8.47	34.35 ± 8.59	
Vegetarian diet duration (years)	5.43 ± 5.05	–	
Education, *n* (%)			0.676
Elementary and secondary	73 (33.3)	80 (35.6)	
Undergraduate	107 (48.9)	97 (43.8)	
Graduate or above	39 (17.8)	45 (20.5)	
Income per month, *n* (%)			0.200
<3,000	42 (19.1)	56 (25.5)	
3,000 ~ 5,000	40 (18.2)	36 (16.4)	
5,000 ~ 8,000	55 (25.0)	59 (26.8)	
>8,000	73 (33.2)	69 (31.4)	
Nondrinker (%)	210 (94.6)	185 (83.3)	<0.001*
Nonsmoker (%)	196 (88.3)	202 (91.0)	0.405
Exercise time (min/w)	119.59 ± 150.96	85.27 ± 123.96	0.183
PSQI sleep score	2.88 ± 2.19	3.25 ± 1.88	0.590
BMI (kg/m^2^)	21.05 ± 2.62	22.49 ± 3.34	<0.001*
WHR	0.82 ± 0.05	0.84 ± 0.05	<0.001*
PBF (%)	26.17 ± 6.53	28.52 ± 5.95	<0.001*
SBP (mm Hg)	108.07 ± 12.76	112.41 ± 14.18	<0.001*
DBP (mm Hg)	70.00 ± 9.52	70.57 ± 10.29	0.628
TC (mmol/L)	4.08 ± 0.76	4.63 ± 0.83	<0.001*
TG (mmol/L)	0.96 ± 0.53	0.91 ± 0.44	0.393
HDL-C (mmol/L)	1.26 ± 0.26	1.37 ± 0.28	<0.001*
LDL-C (mmol/L)	2.55 ± 0.59	2.97 ± 0.68	<0.001*
FG (mmol/L)	4.64 ± 0.67	4.83 ± 0.39	<0.001*
FI (mU/L)	4.90 ± 2.23	5.90 ± 3.03	<0.001*
HOMA-IR	1.03 ± 0.58	1.28 ± 0.70	<0.001*
HOMA-β (%)	93.28 ± 44.61	94.71 ± 60.12	0.672

* Statistical significance. BMI, body mass index; WHR, waist-to-hip ratio; PBF, percent body fat; SBP, systolic blood pressure; DBP, diastolic blood pressure; TC, total cholesterol; TG, triglyceride; HDL-C, high-density lipoprotein cholesterol; LDL-C, low-density lipoprotein cholesterol; FG, fasting blood glucose; FI, fasting insulin; HOMA-IR, homeostasis model assessment of insulin resistance; HOMA-β, homeostasis model assessment of *β* cell function.

### 3.2 Serum metabolomic profiles and differential metabolites of vegetarians

UPLC-MS/MS detected 205 out of 306 metabolites, while the remaining metabolites were not identified due to their concentrations being below the detection limit. The relative abundance of serum metabolite classes in vegetarian and omnivore groups is shown in Table 2. The analysis revealed significant differences in amino acids, fatty acids, indoles, bile acids, benzene ring compounds, benzoic acids, and pyridines.

**Table 2:** Comparison of relative abundance of serum metabolite classes between vegetarians and omnivores.

Class	Vegetarians (*n* = 222)	Omnivores (*n* = 222)	*p*
Carbohydrates	38.809	39.433	0.392
Organic acids	27.684	27.525	0.099
Amino acids	26.604	26.592	0.027*
Fatty acids	5.660	5.171	<0.001*
Carnitines	0.501	0.540	0.136
SCFAs	0.481	0.463	0.148
Indoles	0.061	0.065	<0.001*
Bile acids	0.048	0.043	0.003*
Benzoic acids	0.041	0.036	0.019*
Benzenoids	0.032	0.041	0.002*
Phenylpropanoic acids	0.031	0.033	0.928
Pyridines	0.022	0.032	0.001*
Phenols	0.019	0.018	0.054
Peptides	0.008	0.008	0.417

* Statistical significance. SCFAs, short-chain fatty acids.

PCA, PLS-DA, and OPLS-DA models were constructed to assess the metabolic differences between the vegetarian and omnivore groups (Supplementary Figures 1A–C). The PCA analysis revealed that the first two components explained a relatively low proportion of the variance, suggesting some overlap in the metabolic profiles of these two groups. To further investigate the group separation, the supervised PLS-DA and OPLS-DA methods were applied to refine and analyze the data, confirming distinct metabolic differences between the two groups. The OPLS-DA model, with *R*^2^X = 0.262, *R*^2^Y = 0.518, and Q^2^Y = 0.461, indicated a moderate explanation of the variance in the independent variables (X) and dependent variable (Y). The results of the permutation test (Supplementary Figure 1D) further validated the effectiveness of the OPLS-DA model in discriminating between the two groups.

**Figure 2:**
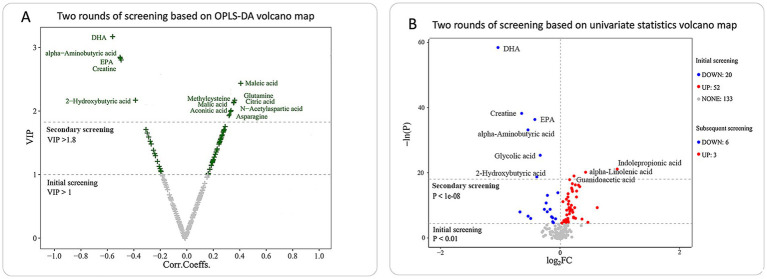
Differential metabolites identified in vegetarians compared to omnivores using two rounds of screening. **(A)** Volcano plot for differential metabolites identified by OPLS-DA between vegetarians and omnivores (initial screening: VIP > 1, secondary screening: VIP > 1.8). **(B)** Volcano plot for differential metabolites identified by univariate analysis in vegetarians vs. omnivores (initial screening: *p* < 0.01, secondary screening: *p* < 1e-08). Significantly increased metabolites in vegetarians (FC > 1 and *p* < 0.01, red dots) and significantly decreased metabolites in vegetarians (FC < 1 and *p* < 0.01, blue dots). FC, fold change; VIP, variable importance in the projection.

**Figure 3:**
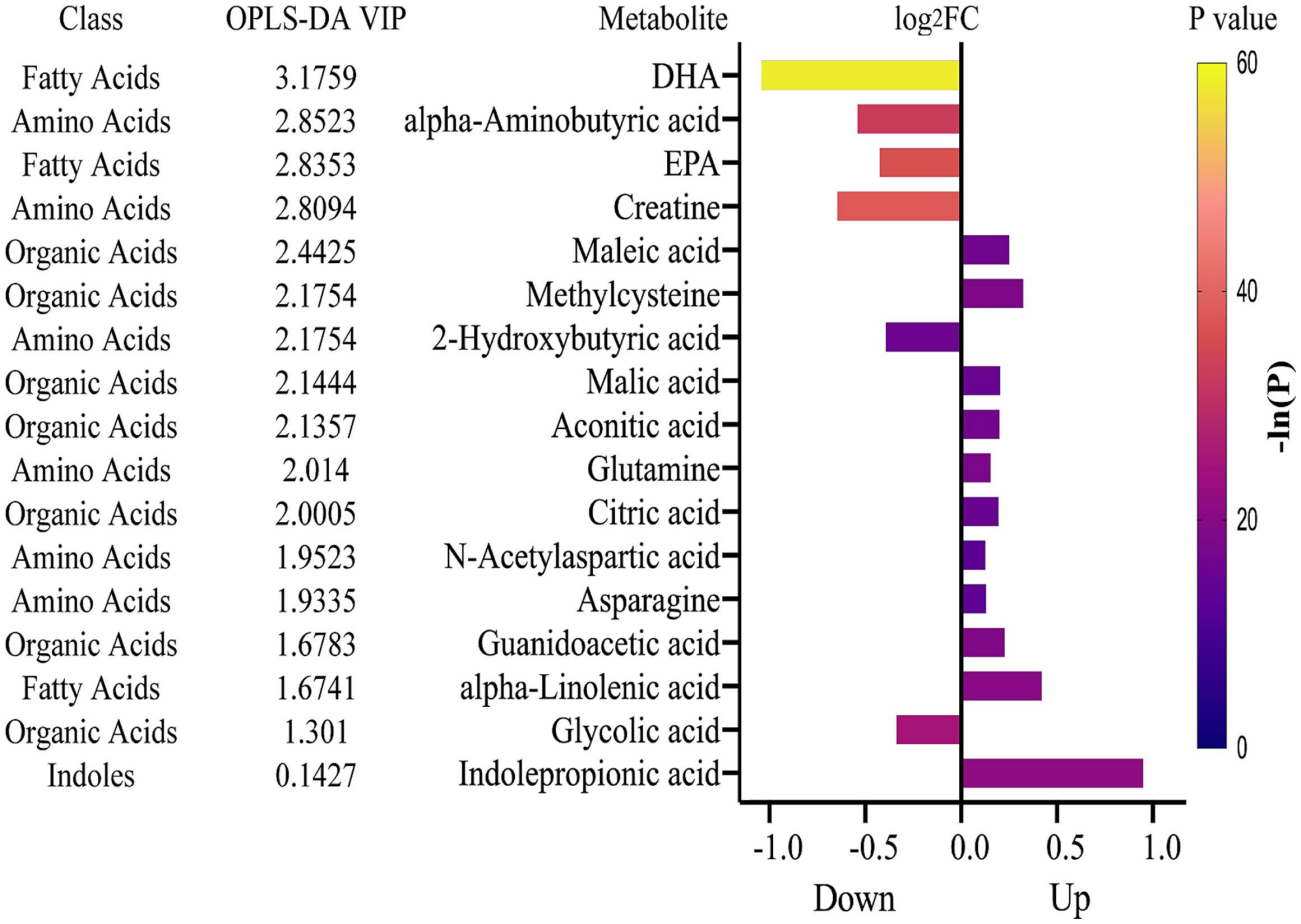
Differential metabolites identified in vegetarians compared to omnivores using the combined results of OPLS-DA and univariate analysis. The bar plot displays metabolite changes, with metabolites ranked by VIP scores from OPLS-DA. FC value for each metabolite (X) is calculated as the ratio of X level in vegetarians to X level in omnivores. Each bar representing an FC value is color-coded according to its corresponding *p*-value. FC values are log2-transformed, and *p*-values are transformed using -ln. FC, fold change; VIP, variable importance in the projection.

In identifying differential metabolites, two rounds of screening were performed using both multivariate and univariate approaches (Figure 2). The first round used thresholds of VIP > 1 (from OPLS-DA) and *p* < 0.01 (from univariate analysis), leading to the identification of 62 metabolites with VIP > 1 and 72 metabolites with *p* < 0.01. The union of these two sets resulted in 83 differential metabolites (Supplementary Table 1). A second round of screening applied stricter criteria, using VIP > 1.8 for the multivariate analysis and *p* < 1e-08 for univariate analysis. This refined selection revealed 13 metabolites meeting the multivariate threshold and 9 metabolites meeting the univariate threshold, resulting in a total of 17 key differential metabolites associated with the vegetarian diet (Figure 3). These metabolites were categorized into four groups: organic acids, amino acids, fatty acids, and indoles. Compared to the omnivore group, 11 metabolites were significantly upregulated in the vegetarian group, including maleic acid, methylcysteine, malic acid, aconitic acid, glutamine, citric acid, N-acetylaspartic acid, asparagine, guanidoacetic acid, *α*-linolenic acid, and indolepropionic acid (IPA). Conversely, 6 metabolites were found to be downregulated in vegetarians, including docosahexaenoic acid (DHA), α-aminobutyric acid, eicosapentaenoic acid (EPA), creatine, 2-hydroxybutyric acid, and glycolic acid. Notably, IPA and maleic acid were the most significantly upregulated metabolites in vegetarians, as evidenced by univariate analysis and OPLS-DA statistics, respectively (IPA: *p* = 6.75e-10; maleic acid: VIP = 2.44). DHA was the most significantly downregulated metabolite in the vegetarian group, with both statistical tests confirming this finding (*p* = 6.76e-26, VIP = 3.18).

To further interpret the biological significance of these differential metabolites, pathway enrichment analysis revealed that these metabolites were primarily involved in six metabolic pathways (*p* < 0.05): Alanine, Aspartate, and Glutamate Metabolism; Glyoxylate and Dicarboxylate Metabolism; Citrate Cycle (TCA Cycle); Arginine and Proline Metabolism; Nitrogen Metabolism; and Glycine, Serine, and Threonine Metabolism (Supplementary Figure 2).

### 3.3 Associations between differential metabolites and cardiometabolic risk factors

**Figure 4:**
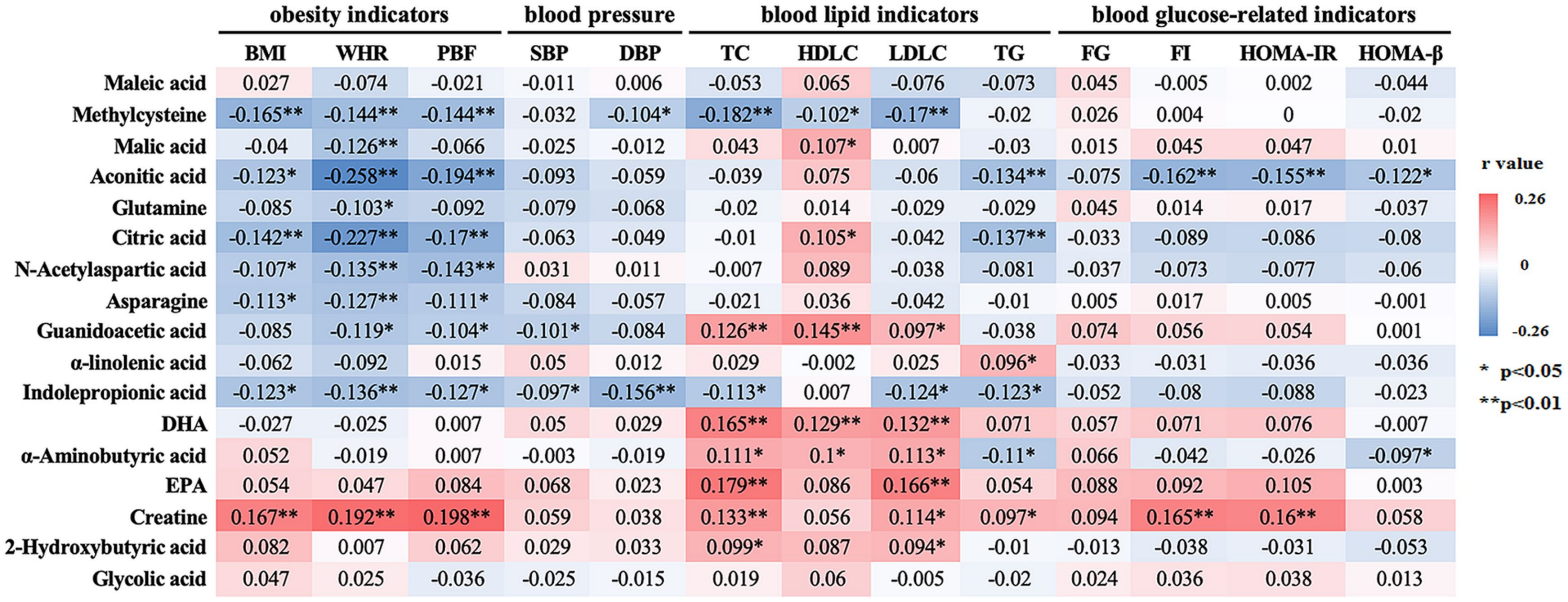
Correlation between differential metabolites and cardiometabolic risk factors. For obesity indicators, the model was adjusted for age, sex, exercise time, alcohol consumption, and dietary pattern; for other indicators, additional adjustments were made for BMI. Correlations were determined using partial correlation analysis. The correlation coefficients are presented and color-coded from red to blue. * *p* < 0.05, ** *p* < 0.01. BMI, body mass index; WHR, waist-to-hip ratio; PBF, body fat percentage; SBP, systolic blood pressure; DBP diastolic blood pressure; TC, total cholesterol; HDL-C, high density lipoprotein cholesterol; LDL-C, low density lipoprotein cholesterol; TG, triglycerides; FG, fasting glucose; FI, fasting insulin; HOMA-IR, homeostasis model assessment of insulin resistance; HOMA-β, homeostasis model assessment of *β*-cell function; DHA, docosahexaenoic acid; EPA, eicosapentaenoic acid.

Partial correlation analysis was employed to explore the intricate relationships between the 17 differential metabolites and key cardiometabolic risk factors, as presented in Figure 4. After adjusting for potential confounding variables, including age, sex, exercise duration, alcohol consumption, and dietary patterns, we identified several metabolites exhibiting distinct patterns of association with obesity indicators. Specifically, metabolites such as methylcysteine, aconitic acid, citric acid, N-acetylaspartic acid, asparagine, and IPA were negatively associated with all three obesity indicators—BMI, WHR, and PBF (*p* < 0.05). In contrast, creatine exhibited a positive association with each of these obesity markers (*p* < 0.05). Further refinement of the analysis, which adjusted for BMI in addition to the aforementioned covariates, revealed that IPA was inversely associated with both systolic and diastolic blood pressure (SBP and DBP) (*p* < 0.05). Methylcysteine, in particular, demonstrated significant negative correlations with three of the four major blood lipid parameters—TC, HDLC, and LDLC (*p* < 0.05). In contrast, metabolites such as guanidoacetic acid, DHA, *α*-aminobutyric acid, and creatine exhibited positive associations with at least three of the four lipid parameters (*p* < 0.05). Additionally, aconitic acid was notably inversely correlated with three of the four blood glucose-related indicators, including FI, HOMA-β, and HOMA-IR (*p* < 0.05).

The associations between these metabolites and cardiometabolic risk factors were further evaluated in multivariate linear regression models, with the same adjustment as partial correlation analysis. These analyses elucidated the complex relationships between these metabolites and obesity indicators, blood pressure, blood lipid levels, and blood glucose markers. A significant inverse relationship was observed between the concentration of methylcysteine and all three obesity indicators. In the T3 group, methylcysteine was associated with a marked reduction in BMI (*β* = −0.886, 95% CI: −1.556, −0.215), WHR (*β* = −0.013, 95% CI: −0.022, −0.003), and PBF (*β* = −1.625, 95% CI: −2.896, −0.354), suggesting that higher levels of this metabolite may be protective against adiposity (*p* < 0.05). Although aconitic acid showed an inverse association with PBF (*β* = −2.343, 95% CI: −3.622, −1.065) in the T3 group (*p* < 0.001), it had a less pronounced effect on BMI and WHR. Conversely, creatine demonstrated a positive association with these obesity markers. Specifically, in the T3 group, creatine was linked with increased BMI (*β* = 1.109, 95% CI: 0.387, 1.831), WHR (*β* = 0.020, 95% CI: 0.009, 0.030), and PBF (*β* = 2.326, 95% CI: 0.963, 3.689), indicating its potential role in promoting fat accumulation (*p* < 0.01) (Table 3).

**Table 3:** Associations between differential metabolites and obesity indicators.

Differential metabolites (μmol/L)	*β* (95%CI) of obesity indicators		
	BMI	WHR	PBF
Methylcysteine			
T1 (1.478 ~ 4.735)	Ref	Ref	Ref
T2 (4.745 ~ 7.189)	−0.727 (−1.381, −0.072)*	−0.008 (−0.017, 0.001)	−0.905 (−2.145, 0.336)
T3 (7.208 ~ 27.961)	−0.886 (−1.556, −0.215)**	−0.013 (−0.022, −0.003)**	−1.625 (−2.896, −0.354)*
*P_trend_*	0.012	0.008	0.013
Aconitic acid			
T1 (25.955 ~ 53.423)	Ref	Ref	Ref
T2 (53.446 ~ 67.249)	−0.775 (−1.436, −0.114)*	−0.012 (−0.021, −0.003)**	−1.610 (−2.850, −0.370)*
T3 (67.296 ~ 147.972)	−0.624 (−1.305, 0.057)	−0.023 (−0.032, −0.013)**	−2.343 (−3.622, −1.065)**
*P_trend_*	0.066	<0.001	<0.001
Citric acid			
T1 (42.435 ~ 117.497)	Ref	Ref	Ref
T2 (117.643 ~ 145.447)	−0.444 (−1.099, 0.211)	−0.008 (−0.017, 0.001)	−1.120 (−2.355, 0.114)
T3 (145.862 ~ 291.268)	−0.546 (−1.217, 0.124)	−0.018 (−0.028, −0.009)**	−1.626 (−2.890, −0.362)*
*P_trend_*	0.113	<0.001	0.013
N-acetylaspartic acid			
T1 (0.217 ~ 0.592)	Ref	Ref	Ref
T2 (0.594 ~ 0.698)	−0.916 (−1.565, −0.268)**	−0.011 (−0.020, −0.002)*	−1.671 (−2.900, −0.442)**
T3 (0.7 ~ 1.432)	−0.877 (−1.539, −0.215)**	−0.014 (−0.023, −0.005)**	−1.542 (−2.796, −0.288)*
*P_trend_*	0.015	0.005	0.025
Asparagine			
T1 (26.377 ~ 59.244)	Ref	Ref	Ref
T2 (59.489 ~ 70.604)	−0.359 (−1.016, 0.299)	−0.010 (−0.019, −0.001)	−1.034 (−2.276, 0.209)
T3 (70.695 ~ 146.187)	−0.261 (−0.941, 0.419)	−0.004 (−0.014, 0.005)	−0.296 (−1.581, 0.989)
*P_trend_*	0.460	0.391	0.694
Guanidoacetic acid			
T1 (0.805 ~ 2.897)	Ref	Ref	Ref
T2 (2.9 ~ 3.734)	−0.059 (−0.711, 0.594)	−0.001 (−0.010, 0.008)	−0.129 (−1.364, 1.106)
T3 (3.736 ~ 9.951)	−0.513 (−1.203, 0.177)	−0.011 (−0.021, −0.002)*	−1.059 (−2.365, 0.247)
*P_trend_*	0.147	0.024	0.120
IPA			
T1 (0.008 ~ 1.366)	Ref	Ref	Ref
T2 (1.388 ~ 2.98)	−0.357 (−1.046, 0.331)	−0.004 (−0.014, 0.006)	−0.674 (−1.979, 0.632)
T3 (2.991 ~ 19.593)	−0.652 (−1.364, 0.059)	−0.011 (−0.021, −0.001)*	−1.208 (−2.557, 0.141)
*P_trend_*	0.072	0.027	0.078
Creatine			
T1 (11.047 ~ 31.003)	Ref	Ref	Ref
T2 (31.065 ~ 48.027)	0.700 (0.035, 1.365)*	0.010 (0.001, 0.019)*	1.114 (−0.142, 2.370)
T3 (48.067 ~ 116.182)	1.109 (0.387, 1.831)**	0.020 (0.009, 0.030)**	2.326 (0.963, 3.689)**
*P_trend_*	0.003	<0.001	0.001

Multivariate linear regression analysis adjusted for age, sex, exercise time, alcohol drink, and diet pattern. T1, low concentration group as reference; T2, medium concentration group; T3, high concentration group. *β* coefficients and 95% confidence intervals are provided. * *p* < 0.05, ** *p* < 0.01. Ref, reference; BMI, body mass index; WHR, waist-to-hip ratio; PBF, body fat percentage; IPA, indolepropionic acid.

In terms of blood pressure, among methylcysteine, guanidoacetic acid, and IPA, only IPA showed strong associations with both SBP and DBP. In the T3 group, IPA was inversely related to both SBP (*β* = −3.838, 95% CI: −6.825, −0.851) and DBP (*β* = −3.579, 95% CI: −5.805, −1.353), suggesting its potential as a regulator of blood pressure (Table 4).

**Table 4:** Associations between differential metabolites and blood pressure.

Differential metabolites (μmol/L)	*β* (95%CI) of blood pressure	
	SBP	DBP
Methylcysteine		
T1 (1.478 ~ 4.735)	Ref	Ref
T2 (4.745 ~ 7.189)	−1.693 (−4.473, 1.087)	−1.989 (−4.063, 0.085)
T3 (7.208 ~ 27.961)	−0.396 (−3.251, 2.459)	−1.905 (−4.035, 0.225)
*P_trend_*	0.852	0.095
Guanidoacetic acid		
T1 (0.805 ~ 2.897)	Ref	Ref
T2 (2.900 ~ 3.734)	−2.214 (−4.952, 0.524)	−2.377 (−4.421, −0.334)*
T3 (3.736 ~ 9.951)	−1.757 (−4.660, 1.145)	−1.109 (−3.276, 1.057)
*P_trend_*	0.227	0.299
IPA		
T1 (0.008 ~ 1.366)	Ref	Ref
T2 (1.388 ~ 2.98)	−1.431 (−4.314, 1.452)	−2.301 (−4.449, −0.152)*
T3 (2.991 ~ 19.593)	−3.838 (−6.825, −0.851)*	−3.579 (−5.805, −1.353)**
*P_trend_*	0.012	0.002

Multivariate linear regression analysis adjusted for age, sex, exercise time, alcohol drink, diet pattern, and BMI. T1, low concentration group as reference; T2, medium concentration group; T3, high concentration group. *β* coefficients and 95% confidence intervals are provided. * *p* < 0.05, ** *p* < 0.01. Ref, reference; SBP, systolic blood pressure; DBP, diastolic blood pressure; IPA, indolepropionic acid.

Several metabolites, such as methylcysteine, citric acid, IPA, and DHA, were significantly associated with lipid profile markers. Methylcysteine demonstrated a consistent inverse relationship with TC (*β* = −0.329, 95% CI: −0.510, −0.148) and LDL-C (*β* = −0.243, 95% CI: −0.384, −0.103) in the T3 group, suggesting its potential role in improving lipid metabolism (*p* < 0.01). Citric acid and IPA displayed a unique pattern, as they did not show significant associations with TC, HDL-C, or LDL-C, but were negatively correlated with TG in the T3 group (citric acid: *β* = −0.141, 95% CI: −0.244, −0.039; IPA: *β* = −0.148, 95% CI: −0.257, −0.038). In contrast, DHA exhibited positive associations with TC (*β* = 0.365, 95% CI: 0.159, 0.572) and LDL-C (*β* = 0.245, 95% CI: 0.085, 0.406), suggesting a role in lipid regulation (*p* < 0.01) (Table 5).

**Table 5:** Associations between differential metabolites and blood lipid indicators.

Differential metabolites (μmol/L)	*β* (95%CI) of blood lipid indicators			
	TC	HDL-C	LDL-C	TG
Methylcysteine				
T1 (1.478 ~ 4.735)	Ref	Ref	Ref	Ref
T2 (4.745 ~ 7.189)	−0.157 (−0.333, 0.019)	−0.017 (−0.075, 0.040)	−0.130 (−0.266, 0.007)	−0.033 (−0.135, 0.069)
T3 (7.208 ~ 27.961)	−0.329 (−0.510, −0.148)**	−0.059 (−0.118, 0.001)	−0.243 (−0.384, −0.103)**	−0.043 (−0.148, 0.062)
*P_trend_*	<0.001	0.047	0.001	0.432
Citric acid				
T1 (42.435 ~ 117.497)	Ref	Ref	Ref	Ref
T2 (117.643 ~ 145.447)	0.038 (−0.139, 0.214)	0.086 (0.029, 0.143)**	−0.038 (−0.175, 0.099)	−0.165 (−0.265, −0.065)**
T3 (145.862 ~ 291.268)	−0.094 (−0.275, 0.087)	0.058 (0.001, 0.117)*	−0.122 (−0.262, 0.018)	−0.141 (−0.244, −0.039)**
*P_trend_*	0.297	0.057	0.087	0.009
Guanidoacetic acid				
T1 (0.805 ~ 2.897)	Ref	Ref	Ref	Ref
T2 (2.900 ~ 3.734)	0.078 (−0.097, 0.252)	0.037 (−0.019, 0.094)	0.033 (−0.103, 0.168)	−0.042 (−0.142, 0.059)
T3 (3.736 ~ 9.951)	0.272 (0.087, 0.457)**	0.081 (0.021, 0.141)**	0.180 (0.037, 0.324)*	−0.009 (−0.116, 0.098)
*P_trend_*	0.004	0.008	0.015	0.854
IPA				
T1 (0.008 ~ 1.366)	Ref	Ref	Ref	Ref
T2 (1.388 ~ 2.98)	−0.051 (−0.237, 0.134)	0.007 (−0.053, 0.067)	−0.045 (−0.189, 0.099)	−0.120 (−0.225, −0.014)*
T3 (2.991 ~ 19.593)	−0.158 (−0.351, 0.034)	0.016 (−0.046, 0.079)	−0.133 (−0.282, 0.016)	−0.148 (−0.257, −0.038)**
*P_trend_*	0.108	0.615	0.082	0.008
DHA				
T1 (0.393 ~ 3.043)	Ref	Ref	Ref	Ref
T2 (3.049 ~ 5.479)	0.222 (0.041, 0.403)*	0.029 (−0.030, 0.088)	0.161 (0.021, 0.302)*	0.135 (0.031, 0.239)*
T3 (5.516 ~ 23.145)	0.365 (0.159, 0.572)**	0.066 (−0.002, 0.133)	0.245 (0.085, 0.406)**	0.132 (0.014, 0.251)*
*P_trend_*	0.001	0.057	0.003	0.031
α-aminobutyric acid				
T1 (6.813 ~ 21.436)	Ref	Ref	Ref	Ref
T2 (21.484 ~ 30.511)	0.177 (−0.006, 0.360)	0.011 (−0.048, 0.070)	0.161 (0.019, 0.303)*	−0.023 (−0.128, 0.082)
T3 (30.539 ~ 79.344)	0.277 (0.088, 0.467)**	0.072 (0.010, 0.134)*	0.209 (0.062, 0.356)**	−0.080 (−0.189, 0.029)
*P_trend_*	0.004	0.022	0.005	0.149
EPA				
T1 (0.224 ~ 0.723)	Ref	Ref	Ref	Ref
T2 (0.724 ~ 0.986)	0.081 (−0.094, 0.256)	−0.029 (−0.086, 0.029)	0.074 (−0.063, 0.210)	0.076 (−0.026, 0.178)
T3 (0.989 ~ 5.919)	0.382 (0.198, 0.566)**	0.059 (−0.001, 0.120)	0.275 (0.131, 0.418)**	0.042 (−0.065, 0.149)
*P_trend_*	<0.001	0.039	<0.001	0.490
Creatine				
T1 (11.047 ~ 31.003)	Ref	Ref	Ref	Ref
T2 (31.065 ~ 48.027)	0.143 (−0.036, 0.322)	0.057 (−0.002, 0.116)	0.047 (−0.093, 0.186)	0.044 (−0.059, 0.147)
T3 (48.067 ~ 116.182)	0.357 (0.161, 0.552)**	0.047 (−0.017, 0.111)	0.240 (0.088, 0.392)**	0.145 (0.033, 0.258)*
*P_trend_*	<0.001	0.160	0.002	0.011
2-hydroxybutyric acid				
T1 (32.239 ~ 114.988)	Ref	Ref	Ref	Ref
T2 (115.157 ~ 167.29)	0.185 (0.009, 0.362)*	0.048 (−0.010, 0.105)	0.138 (0.001, 0.275)*	−0.029 (−0.131, 0.072)
T3 (167.734 ~ 545.478)	0.180 (−0.002, 0.362)	0.055 (−0.004, 0.114)	0.125 (−0.016, 0.266)	−0.054 (−0.159, 0.051)
*P_trend_*	0.050	0.065	0.078	0.310

Multivariate linear regression analysis adjusted for age, sex, exercise time, alcohol drink, diet pattern, and BMI. T1, low concentration group as reference; T2, medium concentration group; T3, high concentration group. *β* coefficients and 95% confidence intervals are provided. * *p* < 0.05, ** *p* < 0.01. Ref, reference; TC, total cholesterol; HDL-C, high density lipoprotein cholesterol; LDL-C, low density lipoprotein cholesterol; TG, triglycerides; IPA, indolepropionic acid; DHA, docosahexaenoic acid; EPA, eicosapentaenoic acid.

Fewer metabolites showed association with blood glucose markers. In the T3 group, aconitic acid was significantly associated with reduced FI (*β* = −0.927, 95% CI: −1.465, −0.388) and HOMA-IR (*β* = −0.214, 95% CI: −0.336, −0.092), indicating its potential to improve insulin sensitivity and glucose metabolism (*p* < 0.01) (Table 6).

**Table 6:** Associations between differential metabolites and blood glucose-related indicators.

Differential metabolites (μmol/L)	*β* (95%CI) of blood glucose-related indicators		
	FI	HOMA-IR	HOMA-β
Aconitic acid			
T1 (25.955 ~ 53.423)	Ref	Ref	Ref
T2 (53.446 ~ 67.249)	−0.591 (−1.114,-0.068)*	−0.131 (−0.250, −0.012)*	−10.010 (−21.331, 1.311)
T3 (67.296 ~ 147.972)	−0.927 (−1.465, −0.388)**	−0.214 (−0.336, −0.092)**	−11.003 (−22.651, 0.644)
*P_trend_*	0.001	0.001	0.062
Creatine			
T1 (11.047 ~ 31.003)	Ref	Ref	Ref
T2 (31.065 ~ 48.027)	0.185 (−0.345, 0.714)	0.037 (−0.083, 0.158)	2.660 (−8.802, 14.121)
T3 (48.067 ~ 116.182)	0.845 (0.267, 1.423)**	0.187 (0.055, 0.318)**	7.533 (−4.976, 20.041)
*P_trend_*	0.004	0.005	0.236

Multivariate linear regression analysis adjusted for age, sex, exercise time, alcohol drink, diet pattern, and BMI. T1, low concentration group as reference; T2, medium concentration group; T3, high concentration group. *β* coefficients and 95% confidence intervals are provided. * *p* < 0.05, ** *p* < 0.01. Ref, reference; FI, fasting insulin; HOMA-IR, homeostasis model assessment of insulin resistance; HOMA-β, homeostasis model assessment of *β*-cell function.

### 3.4 Associations between differential metabolites and dietary components

**Figure 5:**
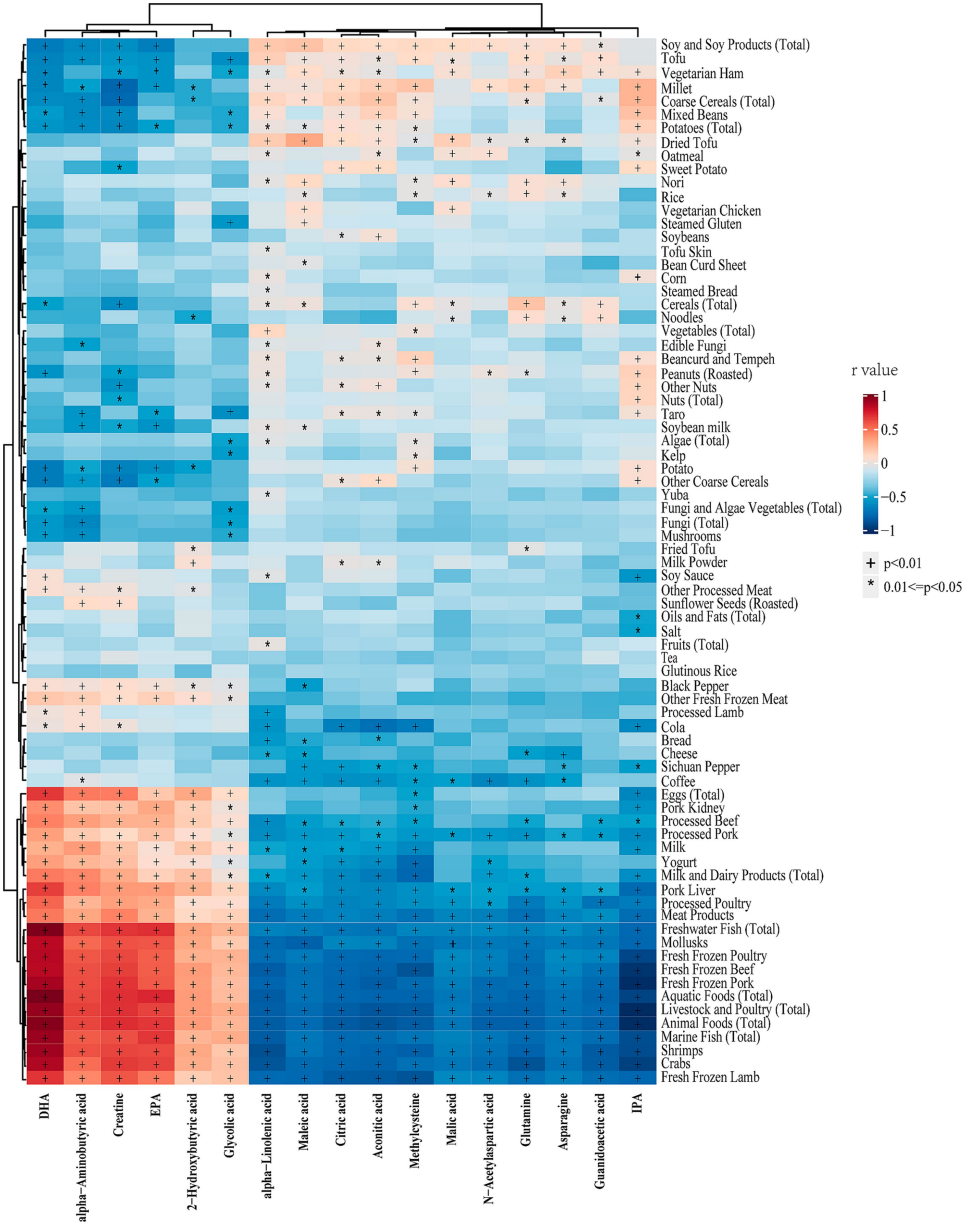
Heatmap of the correlations between differential metabolites and dietary foods in entire cohort. Correlations were determined using Spearman correlation analysis. The colors from red to blue represent the correlation coefficients. DHA, docosahexaenoic acid; EPA, eicosapentaenoic acid; IPA, indolepropionic acid. * *p* < 0.05, † *p* < 0.01.

**Figure 6:**
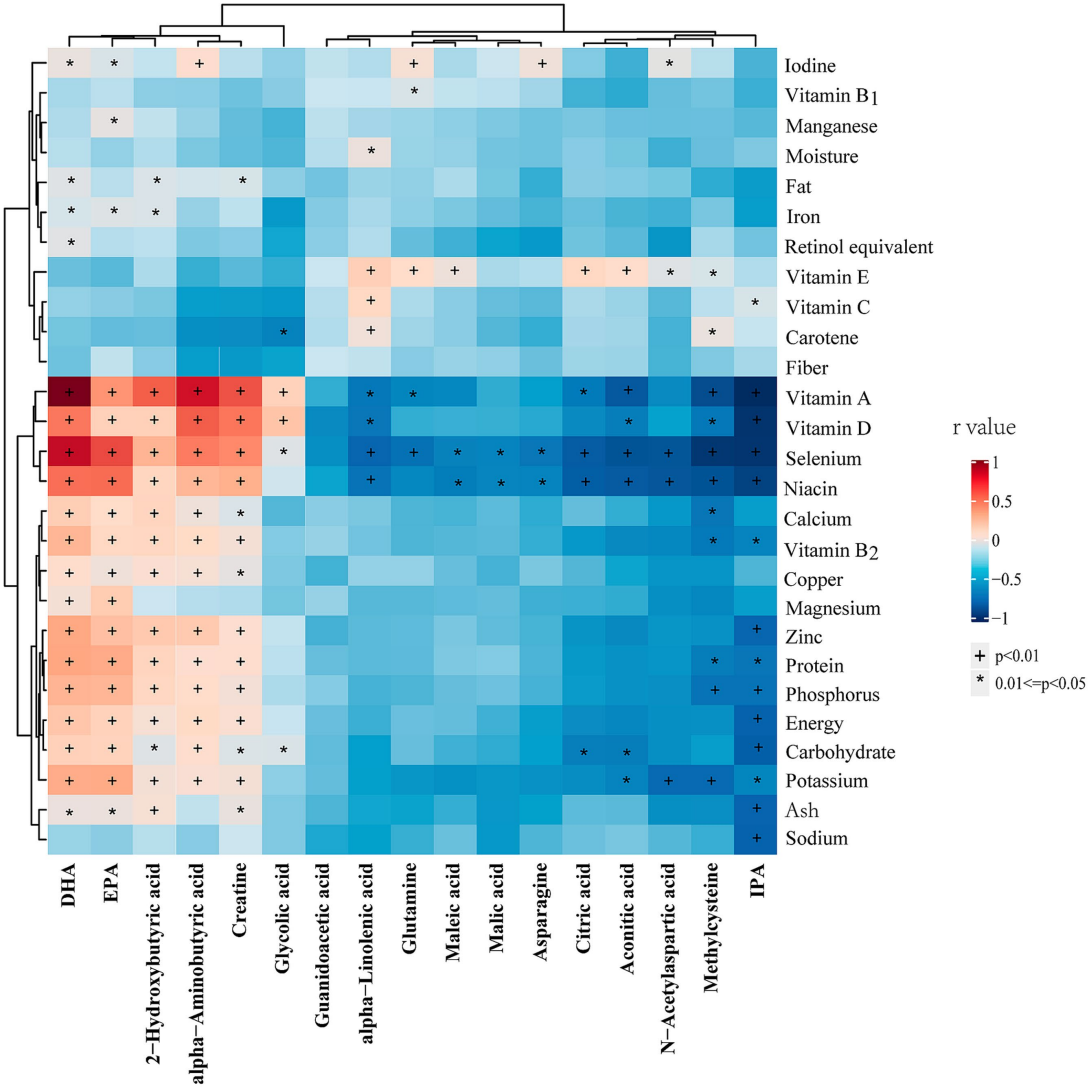
Heatmap of the correlations between differential metabolites and dietary nutrients in entire cohort. Correlations were determined using Spearman correlation analysis. The colors from red to blue represent the correlation coefficients. DHA, docosahexaenoic acid; EPA, eicosapentaenoic acid; IPA, indolepropionic acid. * *p* < 0.05, † *p* < 0.01.

Figure 5 illustrates the correlation analysis between differential metabolites and dietary foods in the entire cohort. The results revealed that all upregulated metabolites in vegetarians were negatively correlated with animal food intake, while all downregulated metabolites exhibited the opposite trend. Additionally, methylcysteine, aconitic acid, citric acid, and maleic acid showed negative correlations with milk or yogurt intake. Notably, IPA displayed a strong negative correlation with animal foods, but a mild positive correlation with millet, coarse grains, mixed beans, and potatoes. Figure 6 presents the correlation analysis between differential metabolites and dietary nutrients in the entire cohort. The upregulated metabolites, such as methylcysteine and IPA, in the vegetarian group were significantly negatively correlated with niacin, selenium, vitamin A, vitamin D, vitamin B2, and several macronutrients. Almost all upregulated metabolites demonstrated a significant negative correlation with niacin and selenium. Conversely, DHA, EPA, *α*-aminobutyric acid, 2-hydroxybutyric acid, and creatine, which were downregulated in the vegetarian group, showed strong positive correlations with niacin, selenium, vitamin A, and vitamin D.

Significant differences in food and nutrient intake were observed between the vegetarian and omnivore groups. To further refine the associations identified in the correlation analysis of the entire cohort, additional analyses were conducted within the vegetarian and omnivore groups to examine the relationships between differential metabolites and dietary foods (Supplementary Figures 3, 4) and nutrients (Supplementary Figures 5, 6). Notably, DHA and EPA showed strong positive correlations with seafood, while maleic acid, malic acid, α-linolenic acid, aconitic acid, and citric acid positively correlated with tofu and soy products. IPA, methylcysteine, N-acetylaspartic acid, and glutamine showed positive correlations with cereals, rice, millet, and peanuts, all observed within the omnivore group (*p* < 0.05). The major source of DHA in vegetarian group is eggs. The significant correlations between differential metabolites and dietary foods observed across the entire cohort, vegetarian group (with meat foods excluded), and omnivore group are summarized in Table 7. Notably, methylcysteine consistently correlated negatively with yogurt intake across all three cohorts (entire cohort: *r* = −0.22, vegetarians: *r* = −0.14, omnivores: *r* = −0.15, all *p* < 0.05). Maleic acid, IPA, and 2-hydroxybutyric acid consistently showed positive correlations with dried tofu, coarse cereals, and total eggs, respectively, across all three cohorts (IPA with coarse cereals entire cohort: *r* = 0.24, vegetarians: *r* = 0.13, omnivores: *r* = 0.16) (2-hydroxybutyric acid with eggs entire cohort: *r* = 0.25, vegetarians: *r* = 0.16, omnivores: *r* = 0.15) (all *p* < 0.05). Meanwhile, the significant correlations between differential metabolites and dietary nutrients observed across the entire cohort and different groups are summarized in Table 8. Consistently, DHA, *α*-aminobutyric acid, and 2-hydroxybutyric acid were positively associated with vitamin A intake across all three cohorts. Of these, DHA showed highest consistent correlation with vitamin A intake (entire cohort: *r* = −0.46, vegetarians: *r* = −0.31, omnivores: *r* = −0.18) (all *p* < 0.05).

**Table 7:** Summary list of correlations between differential metabolites and dietary foods.

Differential metabolites	Associated nutrients	*r*		
		Entire cohort	Vegetarian group	Omnivore group
Maleic acid	Dried Tofu	0.26	0.2	0.19
Methylcysteine	Fresh Frozen Beef	−0.27	/	−0.17
	Fresh Frozen Lamb	−0.27	/	−0.18
	Yogurt	−0.22	−0.14	−0.15
Aconitic acid	Total Animal Foods	−0.27	/	−0.13
	Fresh Frozen Lamb	−0.25	/	−0.14
	Cola	−0.21	−0.14	−0.18
Glutamine	Crabs	−0.27	/	−0.21
	Total Animal Foods	−0.24	/	−0.14
	Total Livestock and Poultry	−0.24	/	−0.13
	Shrimps	−0.24	/	−0.16
	Meat Products	−0.21	/	−0.14
Citric acid	Total Animal Foods	−0.26	/	−0.15
	Fresh Frozen Lamb	−0.25	/	−0.15
N-Acetylaspartic acid	Total Animal Foods	−0.25	/	−0.15
	Crabs	−0.22	/	−0.14
	Coffee	−0.17	−0.14	−0.13
Guanidoacetic acid	Shrimps	−0.26	/	−0.17
IPA	Coarse Cereals Total	0.24	0.13	0.16
DHA	Total Aquatic Foods	0.44	/	0.25
	Total Animal Foods	0.42	/	0.16
	Crabs	0.4	/	0.17
	Shrimps	0.39	/	0.15
	Mollusks	0.38	/	0.2
	Pork Kidney	0.26	/	0.21
α-Aminobutyric acid	Total Livestock and Poultry	0.4	/	0.14
	Total Eggs	0.33	/	0.14
	Pork Liver	0.32	/	0.15
EPA	Total Aquatic Foods	0.56	/	0.26
	Total Animal Foods	0.55	/	0.18
	Shrimps	0.52	/	0.13
	Crabs	0.5	/	0.2
	Mollusks	0.49	/	0.18
	Pork Kidney	0.3	/	0.2
Creatine	Crabs	0.41	/	0.14
2-Hydroxybutyric acid	Total Eggs	0.25	0.16	0.15

Correlations listed in the table were all statistically significant with *p* < 0.05. Spearman’s correlation coefficients (*r*) are provided. IPA, indolepropionic acid; DHA, docosahexaenoic acid; EPA, eicosapentaenoic acid.

**Table 8:** Summary list of correlations between differential metabolites and dietary nutrients.

Differential metabolites	Associated nutrients	*r*		
		Entire cohort	Vegetarian group	Omnivore group
Glutamine	Iodine	0.13	0.14	0.17
DHA	Vitamin A	0.46	0.31	0.18
	Selenium	0.39	0.14	0.15
	Vitamin B_2_	0.21	0.15	0.13
α-Aminobutyric acid	Vitamin A	0.38	0.24	0.18
2-Hydroxybutyric acid	Vitamin A	0.31	0.17	0.2

Correlations listed in the table were all statistically significant with *p* < 0.05. Spearman’s correlation coefficients (*r*) are provided. DHA, docosahexaenoic acid.

## 4 Discussion

Our targeted metabolomic analysis identified 17 key differential metabolites associated with the vegetarian diet, with a particularly notable upregulation of metabolites involved in the TCA cycle, including citric acid, malic acid, maleic acid, aconitic acid, fumaric acid, succinic acid, and isocitric acid. These metabolites play crucial roles in mitochondrial energy production. Although there is no direct evidence linking vegetarian diets to TCA cycle intermediate accumulation (anaplerosis), studies on ketogenic diets—metabolically opposite to plant-based diets—suggest that such diets inhibit anaplerosis (22, 23). This hints at the possibility that vegetarian diets, which rely heavily on carbohydrates, may promote anaplerosis by increasing TCA cycle intermediates. The vegetarian diet, rich in plant-based carbohydrates from fruits, vegetables, and grains, provides a significant carbohydrate source, which supports efficient energy production through mitochondrial processes. Additionally, metabolites like malic acid and citric acid are important for buffering reactive oxygen species (ROS) produced during mitochondrial respiration. This buffering capacity may help explain the reduced oxidative stress observed in vegetarians (24, 25), potentially contributing to their protection against chronic diseases like cardiovascular disease and diabetes. Our findings contrast with a study on high-fat-diet-induced insulin-resistant mice, which reported distinct changes in TCA cycle metabolites associated with metabolic dysfunction and insulin resistance (26). In high-fat-diet-fed mice, serum concentrations of TCA cycle intermediates were significantly reduced. In contrast, the upregulation of these intermediates in vegetarians underscores a clear divergence in metabolic outcomes, highlighting the opposing effects of plant-based and high-fat diets on mitochondrial efficiency. Furthermore, a clinical study on women with obesity showed that combining exercise with a low-calorie diet elevated TCA cycle intermediate, suggesting that such interventions can enhance mitochondrial function (27).

Regarding amino acid metabolism, the elevated levels of glutamine and asparagine in vegetarians suggest alterations in nitrogen metabolism and amino acid catabolism. Glutamine, a key amino acid involved in protein synthesis, immune function, and acid–base balance (28), showed increased concentrations in the vegetarian group. This could indicate an upregulation of protein turnover or enhanced catabolism of non-essential amino acids in vegetarians. Similarly, asparagine, essential for protein synthesis and cellular function (29), showed higher concentrations as well, which also might be related to an increased catabolism of non-essential amino acids. These findings suggest a shift in energy metabolism towards greater dependence on the oxidation of carbohydrates and non-essential amino acids in vegetarians. Furthermore, the downregulation of metabolites such as 2-hydroxybutyric acid and *α*-aminobutyric acid, which are derived from the metabolism of methionine, threonine, and leucine (30, 31), indicates a decreased reliance on the breakdown of essential amino acids. Notably, our findings align with a previous study that identified 2-hydroxybutyric acid as an early biomarker of insulin resistance and glucose intolerance in a non-diabetic population (30). In our study, the reduced levels of 2-hydroxybutyric acid in vegetarians hint at a potential protective effect, which could reduce the risk of insulin resistance or glucose intolerance.

DHA and EPA are essential long-chain *ω*-3 polyunsaturated fatty acids predominantly found in animal-based foods, particularly fish and seafood. As such, the exclusion of meat and seafood from the diet can significantly impact the intake of these crucial fatty acids. The observed lower levels of these metabolites in vegetarians likely reflect a reduced dietary intake of animal- derived *ω*-3 fatty acids, which are vital for maintaining cardiovascular health and modulating inflammatory responses (32). This finding aligns with existing literature, which consistently demonstrates that vegetarians generally exhibit lower levels of DHA and EPA compared to omnivores (33, 34).

However, vegetarians often compensate for this reduced intake of DHA and EPA by increasing their consumption of plant-based ω-3 fatty acids. One of our findings of this study is the elevated levels of *α*-linolenic acid in the serum of vegetarians. *α*-linolenic acid, an essential ω-3 fatty acid, is primarily sourced from plant oils like flaxseed, hemp, and soybean oils (35). These oils are rich in α-linolenic acid, which likely accounts for the higher concentrations observed in individuals following plant-based diets. In contrast, omnivorous diets, which often contain a higher proportion of animal-derived fats, tend to provide lower amounts of this specific fatty acid. Our results align with previous studies, which have also reported increased α-linolenic acid levels in the serum of vegetarians (36). That being said, it is important to note that the conversion efficiency of α-linolenic acid into longer-chain polyunsaturated fatty acids, such as DHA and EPA, can be inadequate to fully compensate for their deficiency in vegetarians (37). For this reason, it is important to emphasize the intake of ALA-rich food to overcome the low conversion efficiency.

Another noteworthy finding was the reduced concentration of creatine in the serum of vegetarians. Creatine, a compound primarily derived from red meat and fish, plays a critical role in energy metabolism, particularly in tissues with high energy demands like muscles and the brain (38, 39). The lower creatine levels in vegetarians are likely attributed to their limited intake of animal products, which are the main dietary sources of this compound. Multiple clinical studies have demonstrated that creatine supplementation can benefit vegetarian athletes by replenishing their creatine levels, boosting energy metabolism, and thereby enhancing overall athletic performance (40). However, an elevation of guanidoacetic acid— a direct precursor to creatine— was observed in vegetarians (41). This suggests that while vegetarians are capable of synthesizing guanidoacetic acid, they may face challenges in efficiently converting it into creatine. This inefficiency in creatine synthesis could be linked to a reduction in methylation processes. The methylation process depends on methyl donors derived from nutrients such as methionine, with S-adenosylmethionine serving as the key molecule for methylation reactions (42). In vegetarians, the lower availability of methionine in their diet, combined with potential vitamin B12 deficiency—a critical cofactor for methionine synthesis and homocysteine metabolism—may lead to a partial impairment of methylation capacity.

In our cohort, vegetarians demonstrated significantly lower BMI, WHR, and PBF compared to omnivores, which is consistent with previous research linking plant-based diets to lower obesity prevalence (43, 44). Further metabolic analysis revealed that methylcysteine and N-acetylaspartic acid were inversely associated with BMI, WHR, and PBF, suggesting their potential role in mitigating fat accumulation. Additionally, aconitic acid and citric acid showed a negative correlation with WHR and PBF. Another noteworthy finding was the inverse relationship between IPA and both SBP and DBP among vegetarians, indicating a potential protective effect of plant-based diets against hypertension (43, 45). Vegetarians also exhibited significantly improved lipid profiles and glucose regulation compared to omnivores, aligning with previous studies associating plant-based diets with better metabolic indices (43, 44). Our metabolomic analysis further revealed that methylcysteine and guanidoacetic acid were significantly correlated with lipid markers. However, it is important to note that the lipid-lowering effects of vegetarianism may come at the expense of reduced bioavailability of DHA and EPA, key *ω*-3 polyunsaturated fatty acids (46). In terms of glucose metabolism, aconitic acid was strongly inversely correlated with both FI and HOMA-IR. Integrating these findings, we identified aconitic acid, citric acid, methylcysteine, and IPA as key serum biomarkers strongly inversely associated with cardiometabolic risk in vegetarians compared to omnivores.

Aconitic acid and citric acid, key intermediates in the TCA cycle, play a crucial role in energy metabolism. A clinical study demonstrated that higher serum levels of both aconitic acid and citric acid were associated with improved insulin sensitivity in adipose tissue (47). Animal studies have shown that serum concentrations of TCA intermediates were significantly reduced in high-fat diet-induced insulin-resistant mice (26). However, there is ongoing debate regarding the beneficial effects of TCA intermediates on metabolic and cardiovascular health, with some studies presenting opposing views (48). Despite these conflicting perspectives, our results indicated that higher serum levels of aconitic acid and citric acid were positively correlated with favorable metabolic outcomes in vegetarians. Further research is needed to fully understand the complex roles of TCA intermediates and their impact on health.

Methylcysteine and its derivatives play a vital role in promoting metabolic health through their potent anti-inflammatory and antioxidant properties. These compounds effectively combat oxidative stress in mice by enhancing glutathione synthesis, reducing the accumulation of ROS, and modulating key inflammatory pathways (49). Additionally, oral treatment of methylcysteine is effective in improving insulin resistance while attenuating metabolic syndrome, inflammation, and oxidative stress in rats fed with fructose rich diet (50). In particular, certain derivatives have been shown to activate NRF2 signaling, a pivotal regulator of cellular antioxidant defenses, while concurrently inhibiting NF-κB and NLRP3 inflammasome activation, thereby enhancing their protective effects against oxidative stress and inflammation (51).

IPA, a gut microbiota-derived metabolite of tryptophan, serves as a key mediator of the interactions between vegetarian diets and the microbiota-host environment (52). Epidemiological studies conducted in Finland, have demonstrated positive correlations between circulating IPA levels and dietary fiber intake, particularly from whole grains (53). In our study, vegetarians exhibited higher serum IPA levels, which were significantly associated with increased cereals, grain, and millet consumption. Growing evidence highlights the relevance of IPA in metabolic diseases, with studies linking its levels to the risk of obesity (54), type 2 diabetes (53), metabolic-associated fatty liver disease (55), and hyperlipidemia (56). Mechanistically, IPA may modulate these metabolic conditions through its involvement in glucose metabolism, insulin sensitivity, lipid homeostasis, inflammatory pathways, and gut microbiota dynamics (57). Additionally, animal studies have demonstrated that IPA exerts beneficial effects on heart function and enhances mitochondrial energy production (58). These findings underscore the potential role of IPA as a critical mediator of the cardiometabolic benefits associated with vegetarian diets.

Our study also explored the relationship between dietary components and metabolite profiles. Notably, metabolites such as methylcysteine, IPA, and maleic acid showed significant correlations with the intake of specific plant-based foods. Methylcysteine, primarily found in foods such as cruciferous vegetables (e.g., broccoli and cabbage) and seeds (e.g., bean and legume) (59, 60), was negatively correlated with animal food intake, which is consistent with its higher concentration in vegetarians. Similarly, IPA, a compound primarily produced by the gut microbiota from dietary tryptophan, was negatively correlated with animal foods but positively correlated with millet, coarse grains, mixed beans, and potatoes, which are staples in vegetarian diets (61, 62). These findings reinforce the idea that plant-based diets, rich in whole grains, legumes, and vegetables, have a distinct metabolic signature that influences health outcomes. In contrast, metabolites like DHA and EPA, which are predominantly found in aquatic foods (particularly fish), were strongly correlated with the intake of these foods (63, 64). This highlights the potential dietary shortfall in vegetarians with respect to *ω*-3 fatty acids. In terms of nutrients, the differential metabolites between vegetarians and omnivores were found to be correlated with niacin, selenium, vitamin A, and vitamin D. This indicates that vegetarians may have a lower intake of certain micronutrients, especially those that are predominantly found in animal-based foods.

The findings of this study suggest that vegetarian diets are associated with distinct serum metabolomic profiles, which contribute to improved cardiometabolic health, as shown in previous research (65). However, the observed downregulation of ω-3 fatty acids and certain fat-soluble vitamins, such as vitamin A and D, in vegetarians highlights the importance of personalized dietary recommendations (32). For vegetarians, especially those adhering to a strict plant-based diet, supplementation with ω-3 fatty acids, like algal oil, and careful monitoring of fat-soluble vitamin levels may be necessary to ensure a well-rounded nutrient intake that supports long-term health (33, 66).

Despite the robust findings, this study has several limitations. First, the cross-sectional design limits the ability to establish causal relationships between diet and metabolic health, and the dietary intake data collected through FFQ are subject to potential biases such as recall errors and inaccuracies in portion estimation (67–69). Therefore, while these findings provide clues for food- and nutrient-related biomarkers, the observed correlations alone cannot establish a direct causal relationship between metabolite changes and specific food or nutrient intake. Longitudinal studies are needed to assess the long-term effects of vegetarian diets on cardiometabolic risk factors and their potential role in disease prevention. Second, we utilized the Q300 commercial metabolomics platform to detect 305 metabolites, which did not include trimethylamine-N-oxide and its associated choline—substances closely linked to vegetarian diets and cardiometabolic diseases. Future studies should incorporate these metabolites to gain a more comprehensive understanding of their potential impact on cardiometabolic health in relation to diet. Third, while this study was conducted in a Chinese cohort, it is important to acknowledge that dietary patterns and metabolic responses to vegetarian diets may vary across different populations. Further studies in diverse populations are needed to explore the generalizability of our findings and determine how cultural and environmental factors influence the metabolic effects of vegetarian diets.

## 5 Conclusion

This study identified distinct serum metabolomic profiles associated with vegetarian diets in a Chinese cohort, which may contribute to a more favorable cardiometabolic risk factor profile. Furthermore, by elucidating differential metabolites linked to dietary intake and metabolic health, our findings provide valuable insights for the development of personalized and culturally appropriate dietary recommendations. We anticipate that this study will deepen the understanding of the metabolic mechanisms underlying the health benefits of vegetarian diets.

## Data Availability

The original contributions presented in the study are included in the article/Supplementary material. Further inquiries can be directed to the corresponding author.

## Supplementary material

SUPPLEMENTARY FIGURE 1 Multivariate analysis of serum metabolomic profiles for the discrimination between vegetarians and omnivores. **(A)** PCA score plot with principal component boxplot. **(B)** PLS-DA score plot with principal component boxplot. **(C)** OPLS-DA score plot. **(D)** Permutation test results showing correlation coefficients.

SUPPLEMENTARY FIGURE 2 Bubble plot of metabolic pathway analysis based on hsa database. The node color is based on its *p* value and the node radius is determined based on their pathway impact values.

SUPPLEMENTARY FIGURE 3 Heatmap of the correlations between differential metabolites and dietary foods in vegetarian group. Correlations were determined using Spearman correlation analysis. The colors from red to blue represent the correlation coefficients. DHA, docosahexaenoic acid; EPA, eicosapentaenoic acid; IPA, indolepropionic acid. * *p* < 0.05, † *p* < 0.01.

SUPPLEMENTARY FIGURE 4 Heatmap of the correlations between differential metabolites and dietary foods in omnivore group. Correlations were determined using Spearman correlation analysis. The colors from red to blue represent the correlation coefficients. DHA, docosahexaenoic acid; EPA, eicosapentaenoic acid; IPA, indolepropionic acid. * *p* < 0.05, † *p* < 0.01.

SUPPLEMENTARY FIGURE 5 Heatmap of the correlations between differential metabolites and dietary nutrients in vegetarian group. Correlations were determined using Spearman correlation analysis. The colors from red to blue represent the correlation coefficients. DHA, docosahexaenoic acid; EPA, eicosapentaenoic acid; IPA, indolepropionic acid. * *p* < 0.05, † *p* < 0.01.

SUPPLEMENTARY FIGURE 6 Heatmap of the correlations between differential metabolites and dietary nutrients in omnivore group. Correlations were determined using Spearman correlation analysis. The colors from red to blue represent the correlation coefficients. DHA, docosahexaenoic acid; EPA, eicosapentaenoic acid; IPA, indolepropionic acid. * *p* < 0.05, † *p* < 0.01.

## Glossary

BMI: Body mass index
WHR: Waist-to-hip ratio
PBF: Percent body fat
SBP: Systolic blood pressure
DBP: Diastolic blood pressure
TC: Total cholesterol
TG: Triglycerides
LDL-C: Low-density lipoprotein cholesterol
HDL-C: High-density lipoprotein cholesterol
FG: Fasting glucose
FI: Fasting insulin
HOMA-IR: Homeostasis model assessment of insulin resistance
HOMA-β: Homeostasis model assessment of *β*-cell function
UPLC-MS/MS: Ultra-performance liquid chromatography–tandem mass spectrometry
FFQ: Food frequency questionnaire
PCA: Principal component analysis
PLS-DA: Partial least squares-discriminant analysis
OPLS-DA: Orthogonal partial least squares-discriminant analysis
VIP: Variable importance in projection
KEGG: Kyoto encyclopedia of genes and genomes
TCA Cycle: Tricarboxylic acid cycle
DHA: Docosahexaenoic acid
EPA: Eicosapentaenoic acid
IPA: Indolepropionic acid
ROS: Reactive oxygen species
