# Supplementary figures and images for: Serum metabolomic signatures of vegetarians relative to omnivores in a Chinese cohort: associations with cardiometabolic risk factors
Source: Front Nutr. 2025 Sep 23;12:1672143. doi: 10.3389/fnut.2025.1672143 (PMC12500696; doi:10.3389/fnut.2025.1672143)

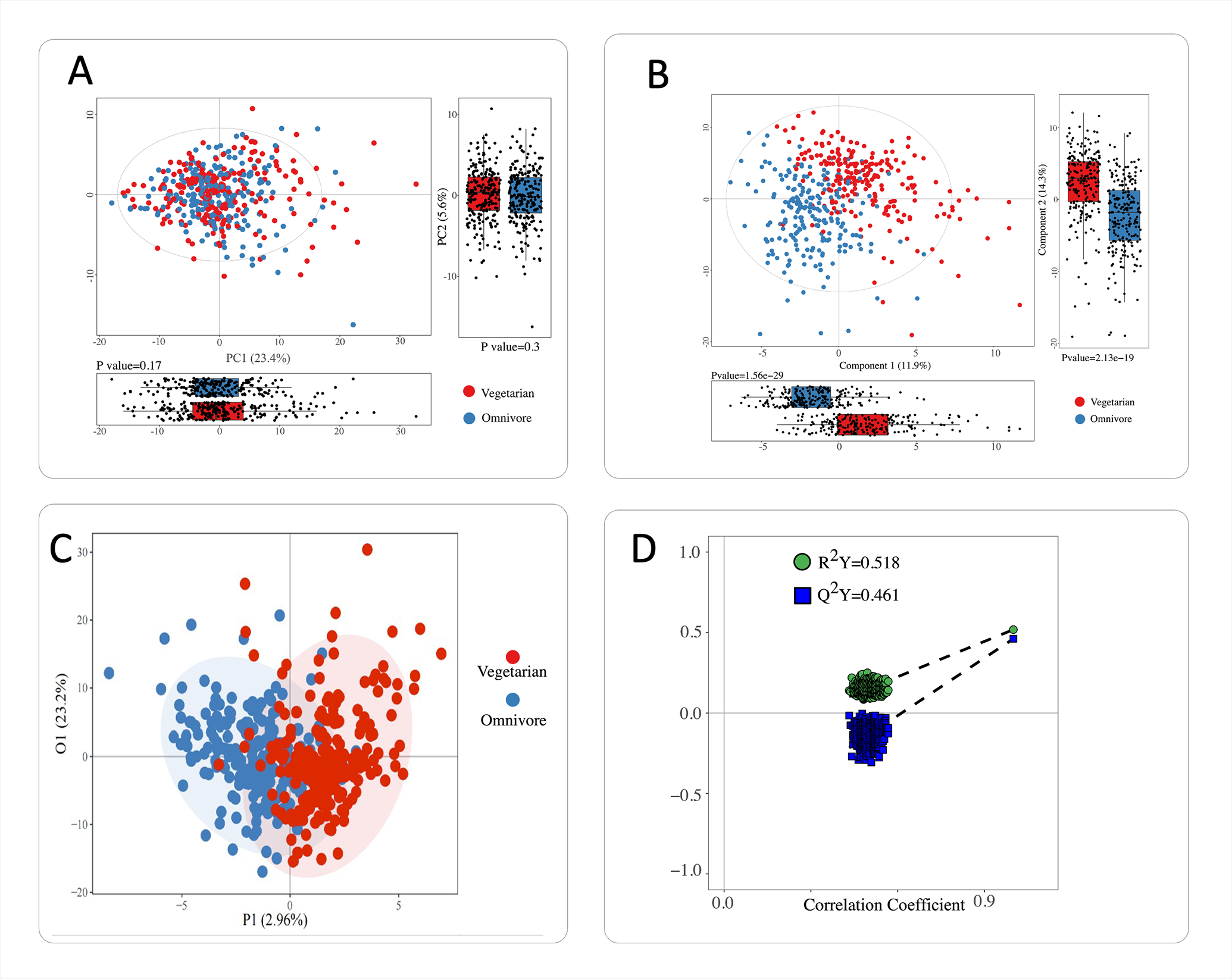

Supplement: SUPPLEMENTARY FIGURE 1 — Multivariate analysis of serum metabolomic profiles for the discrimination between vegetarians and omnivores. (A) PCA score plot with principal component boxplot. (B) PLS-DA score plot with principal component boxplot. (C) OPLS-DA score plot. (D) Permutation test results showing correlation coefficients. [file Image_1.TIF]

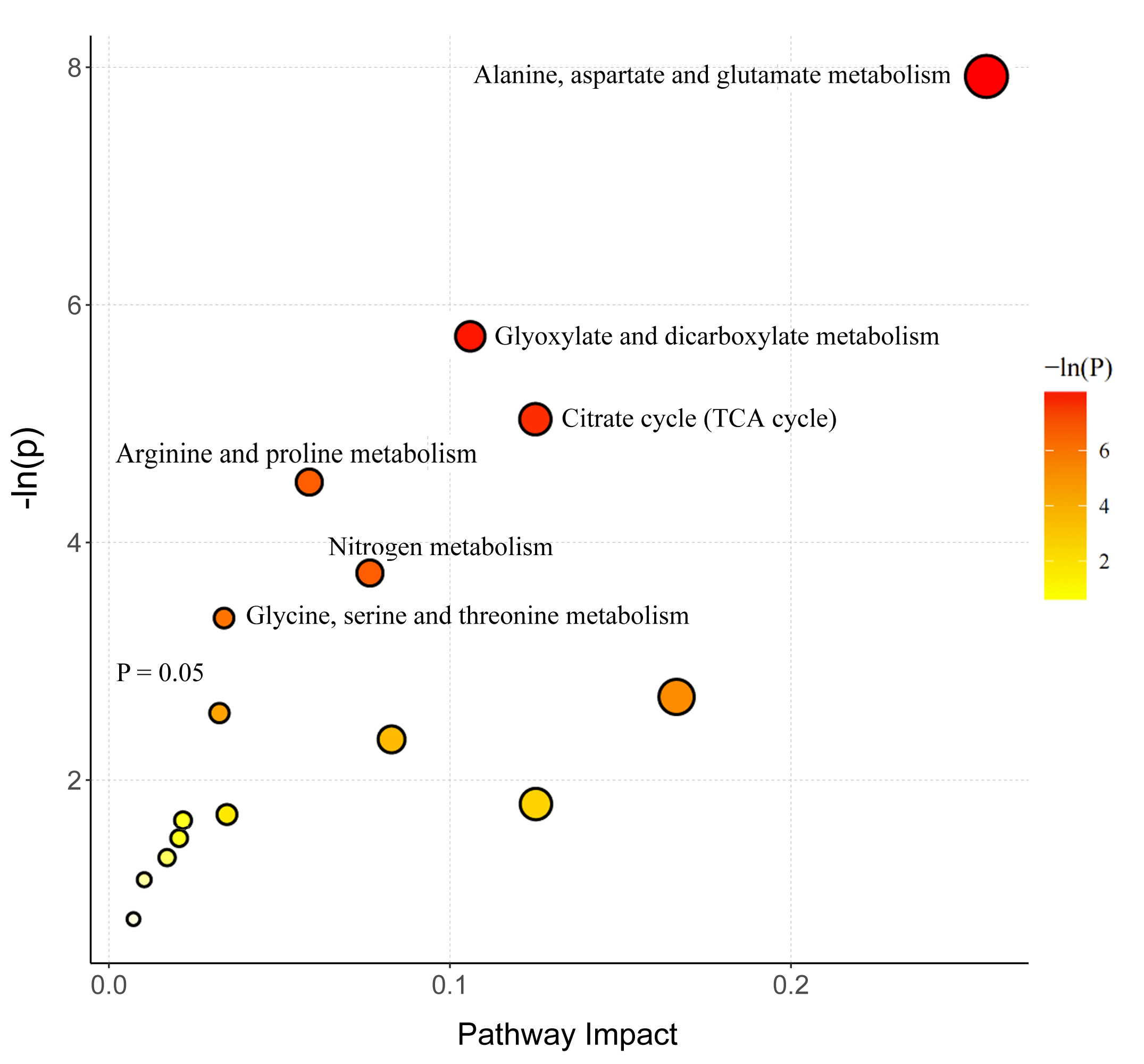

Supplement: SUPPLEMENTARY FIGURE 2 — Bubble plot of metabolic pathway analysis based on hsa database. The node color is based on its p value and the node radius is determined based on their pathway impact values. [file Image_2.TIF]

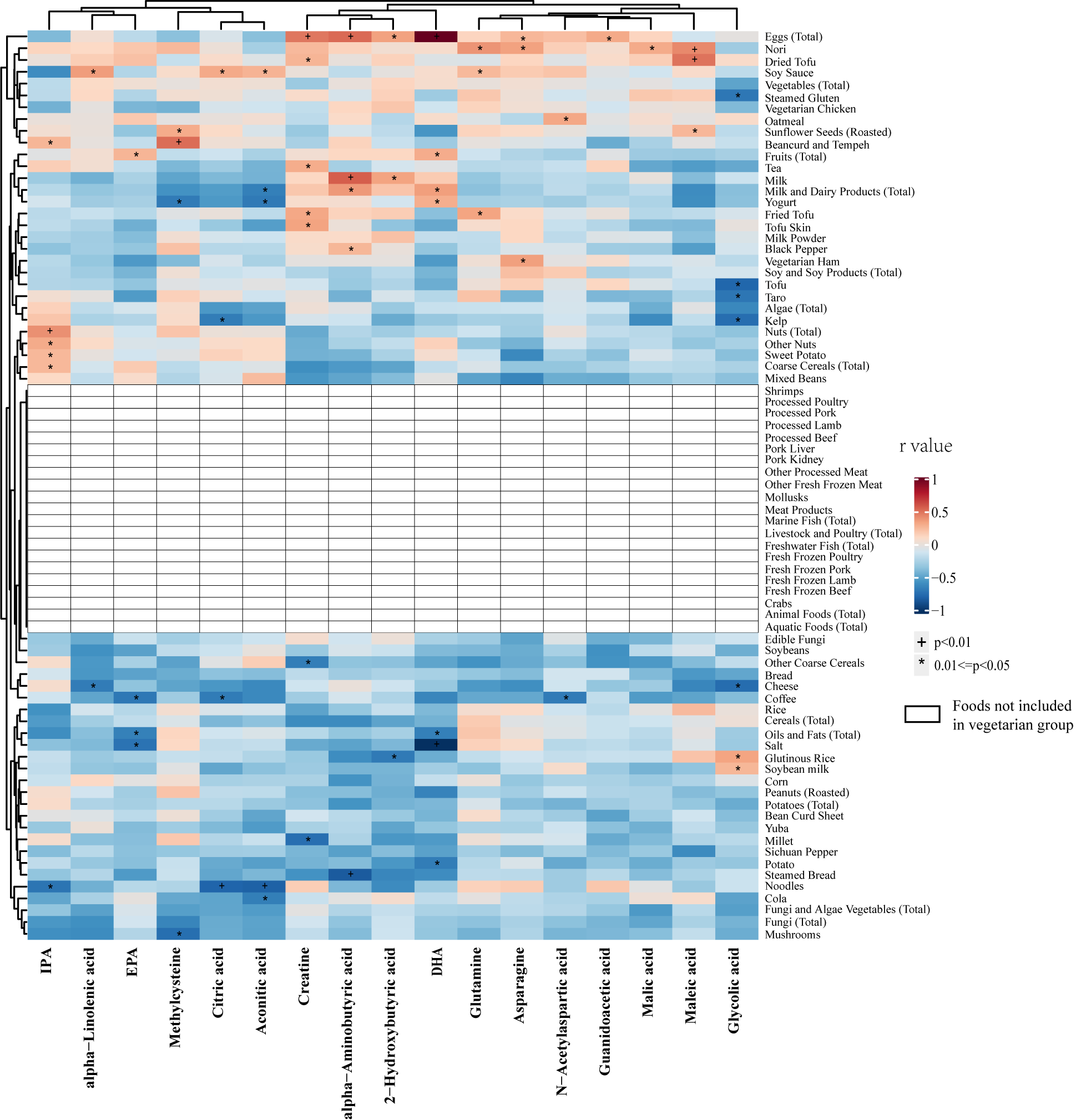

Supplement: SUPPLEMENTARY FIGURE 3 — Heatmap of the correlations between differential metabolites and dietary foods in vegetarian group. Correlations were determined using Spearman correlation analysis. The colors from red to blue represent the correlation coefficients. DHA, docosahexaenoic acid; EPA, eicosapentaenoic acid; IPA, indolepropionic acid. * p < 0.05, † p < 0.01. [file Image_3.TIF]

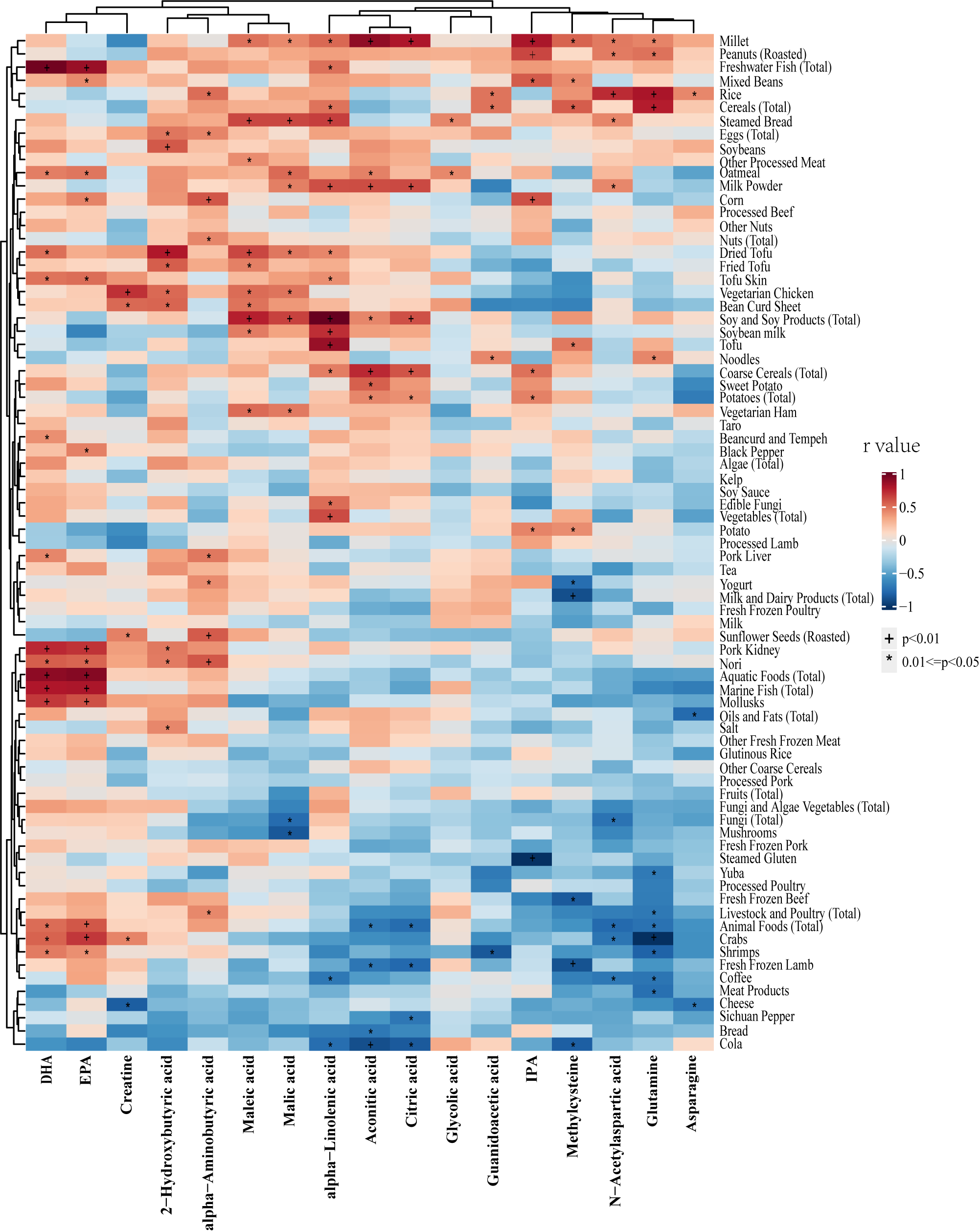

Supplement: SUPPLEMENTARY FIGURE 4 — Heatmap of the correlations between differential metabolites and dietary foods in omnivore group. Correlations were determined using Spearman correlation analysis. The colors from red to blue represent the correlation coefficients. DHA, docosahexaenoic acid; EPA, eicosapentaenoic acid; IPA, indolepropionic acid. * p < 0.05, † p < 0.01. [file Image_4.TIF]

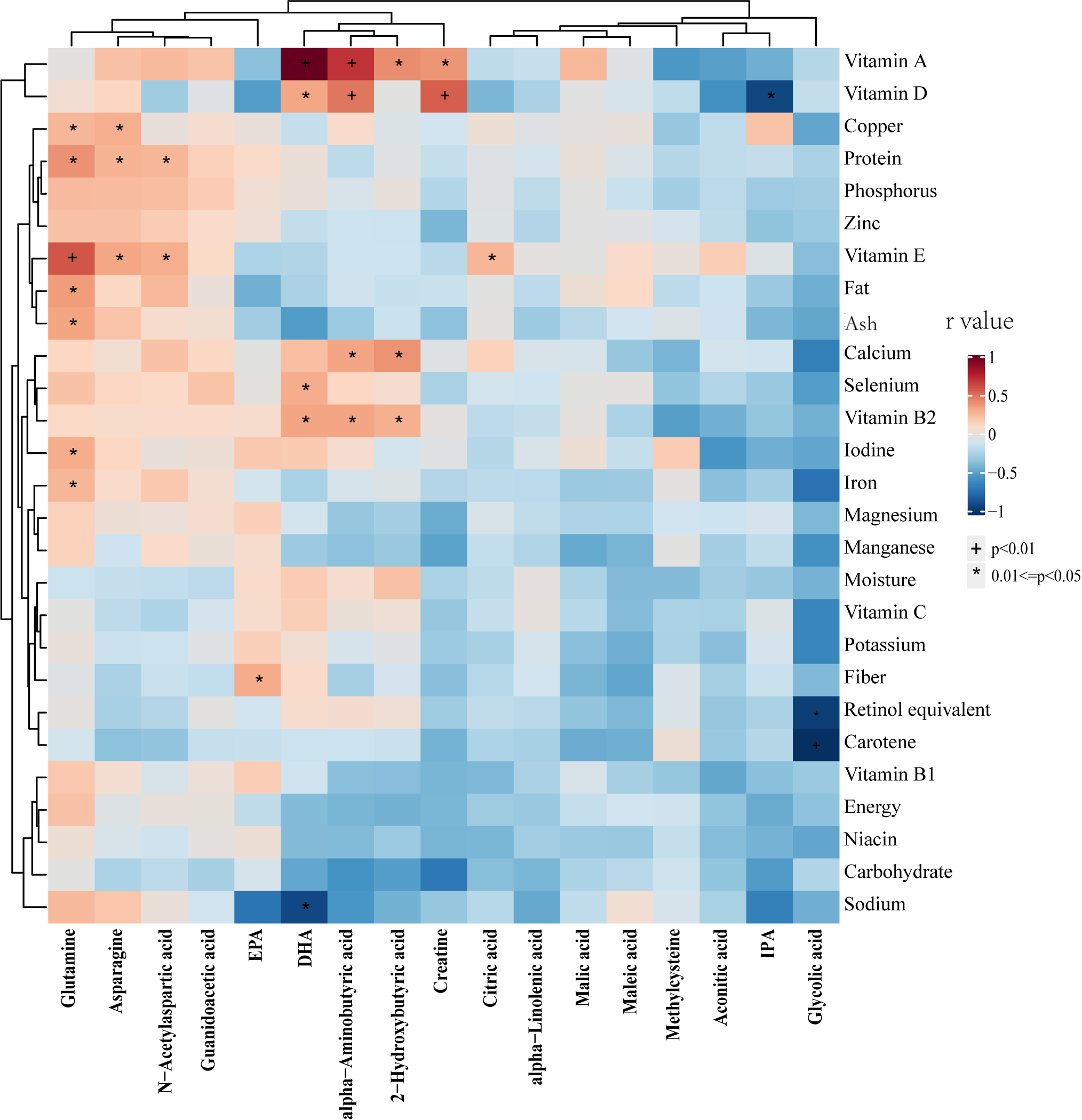

Supplement: SUPPLEMENTARY FIGURE 5 — Heatmap of the correlations between differential metabolites and dietary nutrients in vegetarian group. Correlations were determined using Spearman correlation analysis. The colors from red to blue represent the correlation coefficients. DHA, docosahexaenoic acid; EPA, eicosapentaenoic acid; IPA, indolepropionic acid. * p < 0.05, † p < 0.01. [file Image_5.TIF]

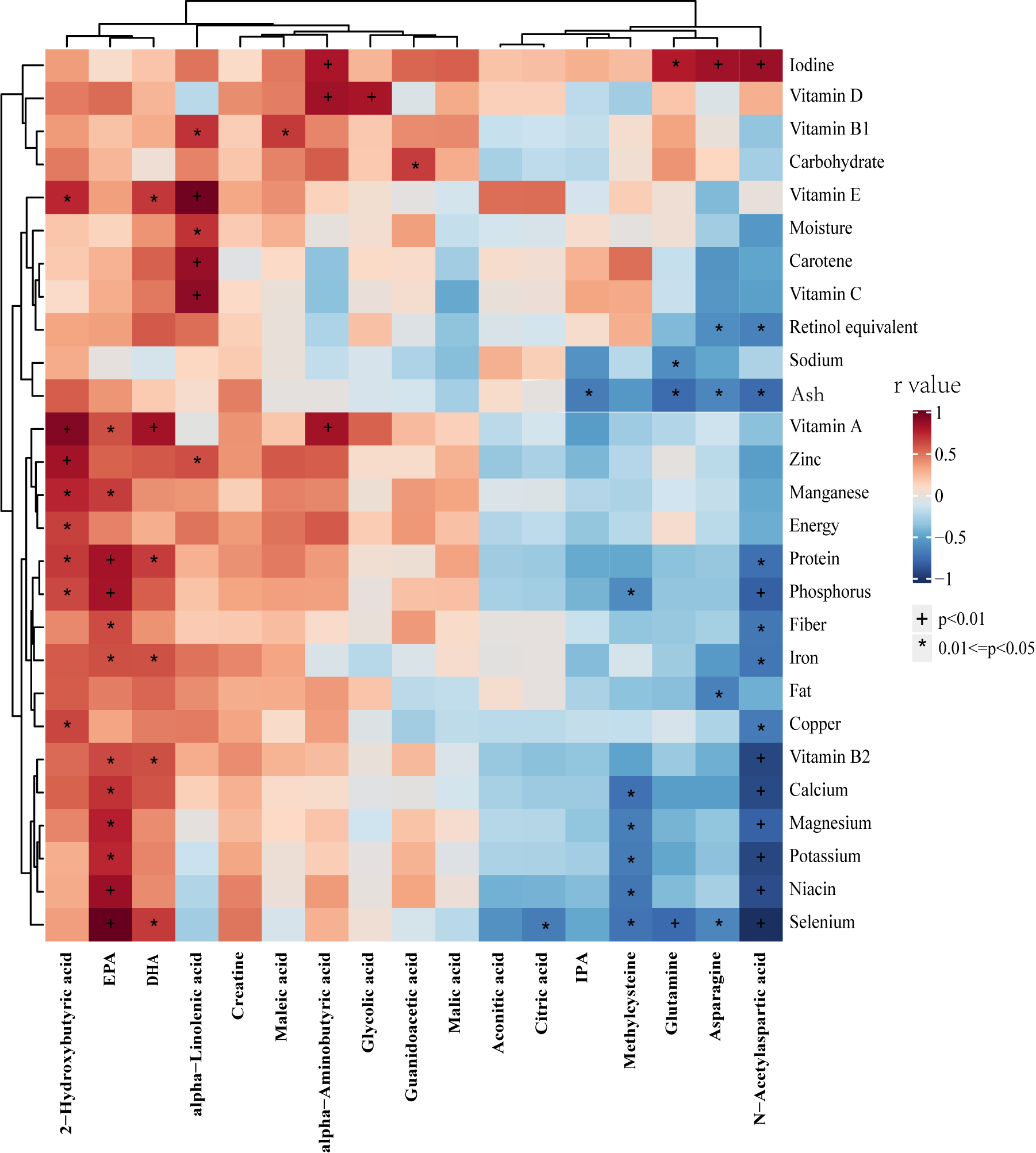

Supplement: SUPPLEMENTARY FIGURE 6 — Heatmap of the correlations between differential metabolites and dietary nutrients in omnivore group. Correlations were determined using Spearman correlation analysis. The colors from red to blue represent the correlation coefficients. DHA, docosahexaenoic acid; EPA, eicosapentaenoic acid; IPA, indolepropionic acid. * p < 0.05, † p < 0.01. [file Image_6.TIF]
