# Supplementary material for: Serum metabolomic signatures of vegetarians relative to omnivores in a Chinese cohort: associations with cardiometabolic risk factors
Source: Front Nutr. 2025 Sep 23;12:1672143. doi: 10.3389/fnut.2025.1672143 (PMC12500696; doi:10.3389/fnut.2025.1672143)
Supplement: Supplementary file 7 [file Table_1.DOCX]

**Supplementary Table 1.** Differential metabolites obtained from initial screening

|  | **Metabolite** | **Class** | **P** | **FC** | **VIP** |
| --- | --- | --- | --- | --- | --- |
| Up-  regulated | Maleic acid | Organic Acids | <0.0001 | 1.1879 | 2.4425 |
|  | Methylcysteine | Amino Acids | <0.0001 | 1.25 | 2.1754 |
|  | Malic acid | Organic Acids | <0.0001 | 1.1496 | 2.1444 |
|  | Aconitic acid | Organic Acids | <0.0001 | 1.1474 | 2.1357 |
|  | Glutamine | Amino Acids | <0.0001 | 1.1104 | 2.014 |
|  | Citric acid | Organic Acids | <0.0001 | 1.1432 | 2.0005 |
|  | N-Acetylaspartic acid | Amino Acids | <0.0001 | 1.0903 | 1.9523 |
|  | Asparagine | Amino Acids | <0.0001 | 1.0925 | 1.9335 |
|  | Fumaric acid | Organic Acids | <0.0001 | 1.1345 | 1.7619 |
|  | Proline | Amino Acids | <0.0001 | 1.1212 | 1.7139 |
|  | GLCA-3S | Bile Acids | <0.0001 | 1.5306 | 1.7106 |
|  | Glycine | Amino Acids | <0.0001 | 1.1380 | 1.695 |
|  | Guanidoacetic acid | Organic Acids | <0.0001 | 1.1698 | 1.6783 |
|  | alpha-Linolenic acid | Fatty Acids | <0.0001 | 1.3377 | 1.6741 |
|  | Succinic acid | Organic Acids | 0.0042 | 1.0645 | 1.6306 |
|  | Erythronic acid | Carbohydrates | <0.0001 | 1.1009 | 1.6174 |
|  | Glycylproline | Peptides | <0.0001 | 1.0291 | 1.5954 |
|  | Linoleic acid | Fatty Acids | <0.0001 | 1.2062 | 1.5713 |
|  | Isocitric acid | Organic Acids | 0.0001 | 1.1048 | 1.5641 |
|  | Methylmalonic acid | Organic Acids | 0.0106 | 1.0592 | 1.556 |
|  | Threonic acid | Carbohydrates | <0.0001 | 1.0949 | 1.5318 |
|  | Gluconolactone | Carbohydrates | 0.0001 | 1.1203 | 1.5143 |
|  | Oxalic acid | Organic Acids | <0.0001 | 1.1046 | 1.5036 |
|  | 2-Hydroxyglutaric acid | Organic Acids | 0.003 | 1.0867 | 1.4856 |
|  | GHCA | Bile Acids | 0.0007 | 1.0760 | 1.4102 |
|  | Pipecolic acid | Amino Acids | 0.0002 | 1.1156 | 1.3924 |
|  | Citrulline | Amino Acids | 0.0002 | 1.0749 | 1.3615 |
|  | GCDCA | Bile Acids | 0.0026 | 1.2825 | 1.3246 |
|  | Pyruvic acid | Organic Acids | 0.0187 | 1.0883 | 1.2804 |
|  | Methylmalonylcarnitine | Carnitines | 0.003 | 1.0617 | 1.2408 |
|  | Alanine | Amino Acids | 0.0021 | 1.0899 | 1.2243 |
|  | Serine | Amino Acids | 0.0081 | 1.0307 | 1.2093 |
|  | Linoleylcarnitine | Carnitines | <0.0001 | 1.2039 | 1.2083 |
|  | Hydroxypropionic acid | Organic Acids | 0.0421 | 1.0666 | 1.1979 |
|  | Ornithine | Amino Acids | 0.0037 | 1.0900 | 1.1925 |
|  | Ribonic acid | Carbohydrates | 0.0002 | 1.1460 | 1.1533 |
|  | Lactic acid | Organic Acids | 0.0572 | 1.0432 | 1.1491 |
|  | Butyric acid | SCFAs | 0.0784 | 1.0586 | 1.0898 |
|  | Ricinoleic acid | Fatty Acids | <0.0001 | 1.0713 | 1.0868 |
|  | Histidine | Amino Acids | 0.0095 | 1.0311 | 1.086 |
|  | N-Acetylserine | Amino Acids | 0.0095 | 1.0476 | 1.023 |
|  | Glyceric acid | Carbohydrates | 0.0177 | 1.0552 | 1.0181 |
|  | GCA | Bile Acids | 0.002 | 1.1907 | 1.0161 |
|  | 4-Hydroxyphenylpyruvic acid | Phenols | 0.0102 | 1.0847 | 1.0009 |
|  | gamma-Linolenic acid | Fatty Acids | <0.0001 | 1.2319 | 0.9967 |
|  | Dihomo-gamma-linolenic acid | Fatty Acids | <0.0001 | 1.1789 | 0.9698 |
|  | Rhamnose | Carbohydrates | 0.0018 | 1.1637 | 0.8674 |
|  | N-Acetylglutamine | Amino Acids | 0.0084 | 1.0155 | 0.8092 |
|  | Myristoleic acid | Fatty Acids | 0.0014 | 1.1641 | 0.7622 |
|  | Malonic acid | Organic Acids | 0.0066 | 1.0834 | 0.7539 |
|  | Dodecanoic acid | Fatty Acids | 0.0069 | 1.3738 | 0.714 |
|  | Isocaproic acid | SCFAs | 0.0075 | 1.0641 | 0.7043 |
|  | 2-Hydroxy-3-methylbutyric acid | Fatty Acids | 0.0055 | 1.0875 | 0.6628 |
|  | Sebacic acid | Fatty Acids | 0.0041 | 1.0401 | 0.6376 |
|  | Cystine | Amino Acids | 0.0057 | 1.0587 | 0.6218 |
|  | 5Z-Dodecenoic acid | Fatty Acids | 0.0005 | 1.1092 | 0.6017 |
|  | 9E-tetradecenoic acid | Fatty Acids | 0.0003 | 1.1535 | 0.5426 |
|  | Oleic acid | Fatty Acids | 0.0062 | 1.0958 | 0.5418 |
|  | IPA | Indoles | <0.0001 | 1.9286 | 0.1427 |
| Down-  regulated | DHA | Fatty Acids | <0.0001 | 0.4853 | 3.1759 |
|  | alpha-Aminobutyric acid | Amino Acids | <0.0001 | 0.6866 | 2.8523 |
|  | EPA | Fatty Acids | <0.0001 | 0.7435 | 2.8353 |
|  | Creatine | Amino Acids | <0.0001 | 0.6383 | 2.8094 |
|  | 2-Hydroxybutyric acid | Organic Acids | <0.0001 | 0.7603 | 2.1754 |
|  | Isovalerylcarnitine | Carnitines | 0.0001 | 0.8300 | 1.7152 |
|  | 2-Methylhexanoic acid | Fatty Acids | <0.0001 | 0.9705 | 1.6599 |
|  | 4-Methylhexanoic acid | Fatty Acids | 0.0125 | 0.9783 | 1.5967 |
|  | 1-Methylnicotinamide | Pyridines | 0.0011 | 0.6874 | 1.5303 |
|  | Phenylacetic acid | Benzenoids | 0.0021 | 0.7086 | 1.4867 |
|  | Phenylacetylglutamine | Amino Acids | 0.0003 | 0.6257 | 1.3886 |
|  | Stearylcarnitine | Carnitines | <0.0001 | 0.8473 | 1.3346 |
|  | Glycolic acid | Organic Acids | <0.0001 | 0.7912 | 1.301 |
|  | Homocitrulline | Amino Acids | 0.0001 | 0.8873 | 1.2126 |
|  | Glutarylcarnitine | Carnitines | <0.0001 | 0.8599 | 1.1339 |
|  | 10Z-Heptadecenoic acid | Fatty Acids | 0.0156 | 0.8545 | 1.0983 |
|  | Propionylcarnitine | Carnitines | 0.1411 | 0.9601 | 1.0644 |
|  | Pentadecanoic acid | Fatty Acids | 0.4144 | 0.9258 | 1.0527 |
|  | Carnitine | Carnitines | 0.0024 | 0.9451 | 0.9815 |
|  | Aminocaproic acid | Amino Acids | 0.0017 | 0.9132 | 0.9222 |
|  | Valine | Amino Acids | 0.0078 | 0.9223 | 0.908 |
|  | 2-Methylbutyroylcarnitine | Carnitines | 0.0003 | 0.8584 | 0.8823 |
|  | Lysine | Amino Acids | 0.0013 | 0.9026 | 0.8257 |
|  | Acetylglycine | Amino Acids | 0.0058 | 0.9141 | 0.4503 |

Abbreviations: GLCA, glycolithocholic acid; GHCA, glycohyocholic acid; GCDCA, glycochenodeoxycholic acid; GCA, glycocholicacid; IPA, indolepropionic acid; DHA, docosahexaenoic acid; EPA, eicosapentaenoic acid; FC, fold change; VIP, variable importance in the projection.
